# Climate Change and Management Impacts on Soybean N Fixation, Soil N Mineralization, N_2_O Emissions, and Seed Yield

**DOI:** 10.3389/fpls.2022.849896

**Published:** 2022-04-27

**Authors:** Elvis F. Elli, Ignacio A. Ciampitti, Michael J. Castellano, Larry C. Purcell, Seth Naeve, Patricio Grassini, Nicolas C. La Menza, Luiz Moro Rosso, André F. de Borja Reis, Péter Kovács, Sotirios V. Archontoulis

**Affiliations:** ^1^Department of Agronomy, Iowa State University, Ames, IA, United States; ^2^Department of Agronomy, Kansas State University, Manhattan, KS, United States; ^3^Department of Crop, Soil, and Environmental Sciences, University of Arkansas, Fayetteville, AR, United States; ^4^Department of Agronomy and Plant Genetics, University of Minnesota, Saint Paul, MN, United States; ^5^Department of Agronomy and Horticulture, University of Nebraska-Lincoln, Lincoln, NE, United States; ^6^Agricultural Center, Louisiana State University, Alexandria, LA, United States; ^7^Department of Agronomy, Horticulture, and Plant Science, South Dakota State University, Brookings, SD, United States

**Keywords:** biological N fixation, soil N mineralization, APSIM, N_2_O emissions, N balance, climate change, weather variability, soybean yield

## Abstract

Limited knowledge about how nitrogen (N) dynamics are affected by climate change, weather variability, and crop management is a major barrier to improving the productivity and environmental performance of soybean-based cropping systems. To fill this knowledge gap, we created a systems understanding of agroecosystem N dynamics and quantified the impact of controllable (management) and uncontrollable (weather, climate) factors on N fluxes and soybean yields. We performed a simulation experiment across 10 soybean production environments in the United States using the Agricultural Production Systems sIMulator (APSIM) model and future climate projections from five global circulation models. Climate change (2020–2080) increased N mineralization (24%) and N_2_O emissions (19%) but decreased N fixation (32%), seed N (20%), and yields (19%). Soil and crop management practices altered N fluxes at a similar magnitude as climate change but in many different directions, revealing opportunities to improve soybean systems’ performance. Among many practices explored, we identified two solutions with great potential: improved residue management (short-term) and water management (long-term). Inter-annual weather variability and management practices affected soybean yield less than N fluxes, which creates opportunities to manage N fluxes without compromising yields, especially in regions with adequate to excess soil moisture. This work provides actionable results (tradeoffs, synergies, directions) to inform decision-making for adapting crop management in a changing climate to improve soybean production systems.

## Introduction

Nitrogen (N) is among the largest factors influencing crop productivity and environmental performance (Cassman and Dobermann, 2022). The cycling of N in the soil–plant-atmosphere continuum is complex and involves many processes including biological N fixation (BNF), plant N uptake, soil N mineralization, and N loss. These processes interact with each other creating tradeoffs and synergies, varying in magnitude and temporal patterns, and are also affected by interactions among genotype, environment, and management—GxExM (e.g., Assefa et al., 2019; de Borja Reis et al., 2021). Environmental sustainability challenges are highly associated with N processes (e.g., N leaching, low BNF). Profitable and environmentally sustainable cropping systems will require alterations in the magnitude of some N processes in certain ways to achieve desired outcomes. Our knowledge of how and in which direction N processes are affected by climate change, weather variability, and management settings are limited. This is because most research work focuses on a single aspect of the system, making it difficult to understand how alterations to part of the system we control—genetics and management—will function in the context of a changing environment including both weather variability and climate change. This knowledge gap needs to be addressed in the context of sustainable intensification of existing cropping systems in changing environments (Hunter et al., 2017).

High seed yields require a high amount of N uptake by the crops and/or partitioning of greater amounts of N to the seeds (Sinclair and de Wit, 1976; Salvagiotti et al., 2008; Cafaro La Menza et al., 2017; Gaspar et al., 2017; Balboa et al., 2018). For example, a soybean seed yield of 2.7 Mg ha^–1^ (global average; FAOSTAT, 2021) requires 208 Kg N uptake ha^–1^, while a high yielding soybean of 5.5 Mg/ha (Cafaro La Menza et al., 2019) requires 423 kg N uptake ha^–1^. The N required by the soybean crop can derive from four sources: BNF, soil organic matter mineralization, residual soil nitrate from previous cropping years, and least commonly N fertilization. The contribution of each source is highly variable across GxExM conditions. Previous research indicated that BNF contributes on average between 50 and 60% of the total N uptake (Ciampitti and Salvagiotti, 2018) while the remaining N is supplied by soil indigenous ammonium and nitrate.

Several agricultural practices have been explored in field experiments to increase crop N uptake and close yield gaps. Examples include changes in sowing dates, cultivars, irrigation, N fertilization, plant arrangements, and results are highly variable depending on the environment (Bender et al., 2015; Moreira et al., 2015; Wegerer et al., 2015; Ortel et al., 2020; Zhao et al., 2020; de Borja Reis et al., 2021; Radzka et al., 2021). Projected soybean yield responses to climate change are highly variable depending on model assumptions and baseline climates (Kothari et al., 2022). Some model-based climate change studies indicate soybean seed yields will decline in future climates scenarios (Jin et al., 2017; Schauberger et al., 2017; Zabel et al., 2021) due to a 1.5°C temperature increase by 2050 (Intergovernmental Panel on Climate Change [IPCC], 2018) and changes in precipitation patterns. Rising temperature negatively impacts seed yield by accelerating crop development, but it can also increase soil N mineralization (De Valpine and Harte, 2001; Turner and Henry, 2010). An increase in drought and flooding events can potentially affect BNF more than soil N supply (Purcell et al., 2004; Pasley et al., 2020). The net impact of climate change on sustainability metrics such as N balance (aboveground N derived from BNF minus seed N removal, Collino et al., 2015; Santachiara et al., 2017) remains unknown.

Climate change, crop improvement, and agronomic advances are not incremental but continuous. In the context of these changes, interannual weather variability is large and management interventions must be dynamic to maximize sustainability for specific weather conditions and regions (Iqbal et al., 2018). A simultaneous evaluation of soil–plant N processes and how they are affected by GxExM alterations would improve our understanding of the complex agronomic system while will facilitate systems thinking and conceptualization of solutions to enhance productivity and environmental performance in soybean-based cropping systems. Therefore, we performed a GxExM simulation experiment across 10 soybean production regions in the United States using the Agricultural Production Systems sIMulator–APSIM (Holzworth et al., 2014). The use of cropping systems modeling is necessary because system-level assessments are limited by experimental data. Key N processes such as BNF and mineralization are impossible to measure at high temporal and spatial resolution or estimated for future climate scenarios. Furthermore, past work has demonstrated that the APSIM model can simulate well several processes of the system (soil N dynamics, BNF, crop N uptake, and yields) in a range of conditions and management settings in the United States (Archontoulis et al., 2014, 2020; Puntel et al., 2016; Ebrahimi-Mollabashi et al., 2019; Martinez-Feria et al., 2019; Pasley et al., 2020, 2021). Our objectives were to:

1. Create a systems understanding of how ecosystem N dynamics, including soil N mineralization, BNF, crop N uptake, soil nitrate pool size, and N_2_O emissions, vary across United States soybean production as a function of GxExM interactions.
2. Separate the contribution of weather variability (uncontrollable factors) from crop and soil management practices (controllable factors) to understand potential interventions needed to increase productivity and environmental performance.
3. Quantify the impact of climate change on productivity and key sustainability metrics including N balance (BNF–seed N removal), N_2_O emissions, and seed yield.

## Materials and Methods

### Study Locations and Weather

**FIGURE 1:**
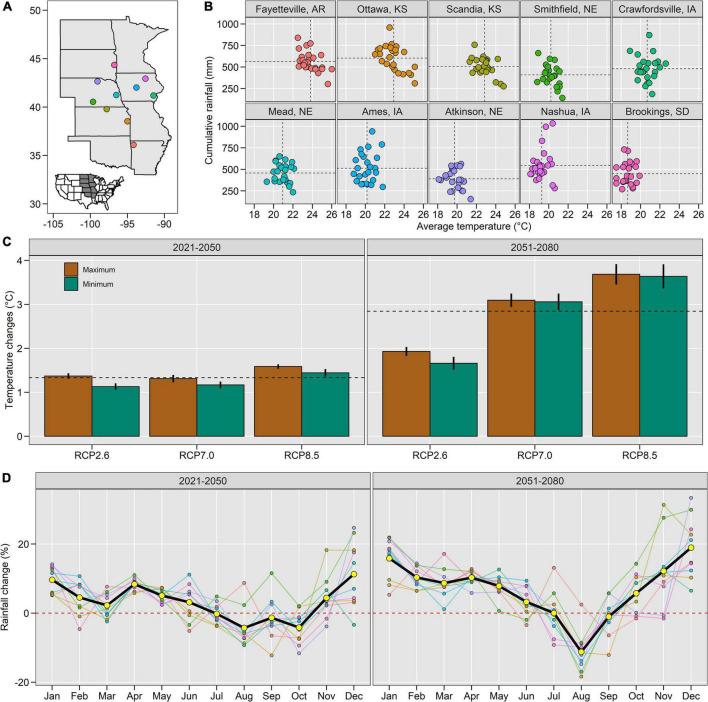
Spatial distribution of experimental locations **(A)**, year-to-year (1995–2020) variability of average temperatures and cumulative rainfall from May to September (horizontal and vertical dashed lines indicate the average values) **(B)**, average temperature changes in relation to the baseline for 2021–2050 and 2051–2080 under RCP2.6, RCP7.0, and RCP8.5 (vertical bars represent the standard deviation from the 10 locations and horizontal dashed lines represent the average change) **(C)**, monthly rainfall changes in relation to the baseline (black lines indicate averages from five global circulation models, three RPCs and 10 locations, while colored lines represent the variability across the 10 locations) **(D)**.

**FIGURE 2:**
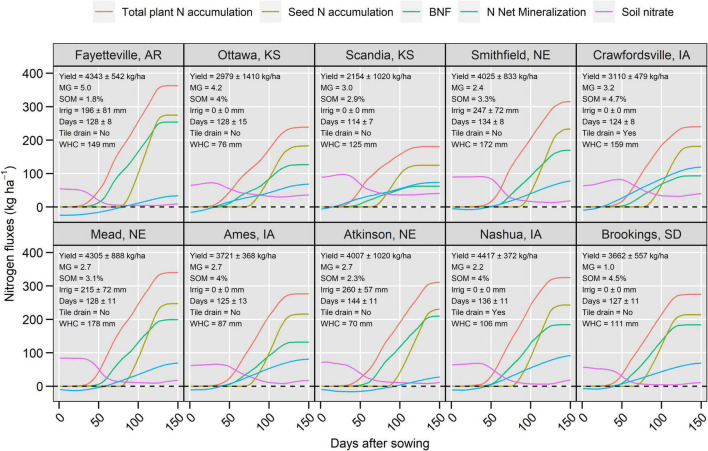
Soil-plant N dynamics including plant and seed N accumulation, biological N fixation (BNF), soil N net mineralization and soil nitrate (0–120 cm) for all locations. The values are averages over 25-year simulations. Inset top-left panels indicate soybean 25-year average seed yield (Yield), maturity group (MG), soil organic matter (SOM, 0–30 cm), irrigation applied during the crop season (Irrig), number of days to physiological maturity (Days), existence of subsurface tile drainage (Tile drain) and soil water holding capacity to 120 cm (WHC). The N balance is illustrated in Supplementary Figure 2. The N_2_O emissions fluxes are illustrated in Supplementary Figure 4.

We performed a simulation experiment across 10 locations in the United States (Figure 1A). The locations were selected to capture different production situations (i.e., soybean maturity groups, water management, and soil and weather conditions, Figures 1, 2). Three locations were rainfed with a water table depth below 3.5 m (Kansas and South Dakota locations), four locations were irrigated with water tables below 3.5 m (Nebraska and Arkansas), one location was rainfed with a shallow water table at about 1.2 m (Ames, IA, United States), and two locations were rainfed with the shallow water table at about 1.2 m and subsurface drainage systems at 1.1 m (Nashua and Crawfordsville, IA, United States). Water table depth data were derived from field measurements and SSURGO (Soil Survey Staff, 2019).

Historical weather data (1995–2020) were retrieved from local weather stations. From May to September, the average temperatures ranged from 18.7 to 23.8°C and rainfall from 390 to 604 mm (Figure 1B). Bias-corrected future climate projections were retrieved from five global circulation models: UKESM1-0-LL, MRI-ESM2-0, MPI-ESM1-2-HR, IPSL-CM6A-LR, and GFDL-ESM4 (Müller et al., 2021; Zabel et al., 2021) and included three representative concentration pathway combinations (RCP2 0.6, RCP 7.0, and RCP 8.5). Bias correction was implemented by using the linear scaling method (Teutschbein and Seibert, 2012), which consists of applying a monthly “delta” correction factor based on the differences between observed and simulated present-day values. On average across all 15 climate scenarios, these projections estimated a 1.3 and 2.8°C increase in the average temperature for the 2020–2050 and 2050–2080 periods, respectively (Figure 1C), and about a 6% increase in spring rainfall and nearly 3% decrease in summer rainfall (Figure 1D).

### APSIM Model Set Up, Calibration, and Evaluation

The APSIM software is an advanced simulator of farming systems (Holzworth et al., 2014). The model simulates crop growth and development of several crops (including rotations), soil water balance, nitrogen, and carbon cycling and contains various management rules such as tillage and subsurface drainage. The soybean crop model simulates biomass production based on a combined radiation and water use efficiency concept and BNF as a function of crop growth rate (crop stage-specific value), which is mediated by drought and excess moisture stresses (Robertson et al., 2002; Pasley et al., 2021). The soil N model simulates soil carbon mineralization, immobilization, nitrification, denitrification, nitrous oxide emissions, and N leaching. The model simulates N mineralization as a function of soil carbon, soil C:N ratio, temperature, and moisture by layer (Probert et al., 1998). The decomposition of crop residue can increase or decrease net mineralization depending on the amount and the CN ratio of the stover (Probert et al., 2005; Archontoulis et al., 2016). The denitrification (and N_2_O emissions) in the model is favored by high soil moisture, temperature, inorganic N, and carbon availability (Huth et al., 2010; Thorburn et al., 2010). The model assumes that crop N uptake can derive from soil inorganic N or BNF, with the soil nitrate being the first priority due to the lower energetic cost (Herridge et al., 2001; Robertson et al., 2002; Chen et al., 2016). For additional information, refer to www.apsim.info.

The APSIM model has been extensively validated in many agroecosystems around the world for the simulation of crop growth, soil water, and soil nitrogen and carbon fluxes (Mohanty et al., 2012; Chen et al., 2016; Battisti et al., 2017; Gaydon et al., 2017; Wu et al., 2019; Archontoulis et al., 2020). Here we used a well-calibrated APSIM version 7.9 (Archontoulis et al., 2020), which has been tested across many high-temporal resolutions, multifaceted, and multi-location datasets in the US Midwest, United States. This version includes algorithms to simulate excess moisture stress on root depth (Ebrahimi-Mollabashi et al., 2019), and on plant growth, development, and BNF (Pasley et al., 2020). Additional studies have verified APSIM capacity in simulating N loss (Malone et al., 2007; Dietzel et al., 2016; Martinez-Feria et al., 2016, 2019; Pasley et al., 2021) and soil N mineralization in the United States Corn Belt (Archontoulis et al., 2014; Puntel et al., 2016). For the simulation of soil water, we used the SWIM3 module available in APSIM (Huth et al., 2012) which uses Richard’s equation and enables the simulation of shallow water tables (Ebrahimi-Mollabashi et al., 2019).

In this study, we further tested the capacity of the model to simulate soil–plant N dynamics before its application to explore climate and management impacts. Experimental data covering high and low soybean yielding environments and maturity groups from 1 to 6 (Supplementary Table 1) were used to develop cultivar coefficients (Supplementary Table 2) with no further changes to the crop or soil models. Soil profile input values were derived from SSURGO (Soil Survey Staff, 2019) or measured data when available and are provided in Supplementary Table 3. Overall, the model proved robust and accurate in simultaneously simulating crop–soil N dynamics across the 10 study locations (Supplementary Figure 1 and Supplementary Table 4).

### Baseline Simulation Conditions

For the baseline simulation, we ran the model for 25 years (1995–2020). Model initial conditions were reset every year on January 1 and were similar among locations to facilitate comparison. Initial conditions included total inorganic N in the profile (75 kg N ha^–1^), maize stover on the surface (4,500 kg ha^–1^ with a C:N ratio of 70), and soil water at field capacity. At each location, we included a moderate tillage event on April 1, with 20% of the surface residue being incorporated to a 20 cm depth. The sowing date was variable per year and per location following USDA-NASS 50% sowing progress (NASS, 2020). Cultivars were site-specific and ranged from maturity group 1 (South Dakota) to 5 (Arkansas, Supplementary Table 2). Plant density ranged from 25 to 32 plants m^–2^ (depending on the location) and row spacing was 0.76 m for all locations except Nashua, IA, United States, which was 0.46 m.

### GxExM Scenarios

Using the well-tested APSIM model for each location, we simulated 22 scenarios (each with 25 weather-years) to create different GxExM conditions (Table 1). The scenarios accounted for climate change (see # 1–3; Table 1), N management strategies (4–7), residue management and quality (8–12), plant management (13–16), cultivar seed protein (17–18), soil organic carbon (19–20), and water management (21–22).

**TABLE 1:** GxExM scenarios assessed using APSIM.

No	Scenario	Acronym	Changes compared to the baseline
1	Rainfall change	Rain change	Relative changes in rainfall (%) based on future climate projections (Figure 1 and Supplementary Table 5)
2	Temperature change	Temp change	Changes in maximum and minimum temperatures (°C) based on future climate projections (Figure 1 and Supplementary Table 5)
3	Rainfall and temperature changes	Rain*Temp	Scenario 1 + Scenario 2
4	Early spring N fertilization	Fer spring	Application of 30 kg N ha^–1^ (DAP or MAP fertilizer)^1^
5	R3-stage N fertilization	FerR3	Application of 60 kg N ha^–1^ (urea fertilizer)
6	More soil leftover N	+ LeftoverN	150 instead of 75 kg N/ha initial N
7	Less soil leftover N	−LeftoverN	38 instead of 75 kg N/ha initial N
8	High residue CN ratio	+ ResCN	150 instead 70 residue CN ratio
9	Low residue CN ratio	−ResCN	35 instead of 70 residue CN ratio
10	More crop residue	+ Residue	9,000 instead of 4,500 kg ha^–1^ residue
11	Less crop residue	−Residue	1,000 instead of 4,500 kg ha^–1^ residue
12	Full tillage	Full tillage	90% instead of 20% residue incorporation to 20 cm depth
13	High sowing density	+ Density	10 plants/m^2^ increase from baseline
14	Low sowing density	−Density	10 plants/m^2^ decrease from baseline
15	Early sowing date	Early sow	12 days earlier sowing than baseline
16	Late sowing date	Late sow	12 days later sowing than baseline
17	High seed protein	+ Seed protein	39.6% instead of 37.1% critical seed protein^2^
18	Low seed protein	−Seed protein	34.6% instead of 37.1% critical seed protein^2^
19	More initial soil organic carbon	+ SOC	15% increase in soil organic carbon
20	Less initial soil organic carbon	−SOC	15% decrease in soil organic carbon
21	Irrigation	Irrig	Irrigation between R1 and R7 when soil water falls below 50% PAW^3^
22	Tile drainage	Tile drainage	Model subsurface drainage function enabled

*^1^DAP, diammonium phosphate; MAP, monoammonium phosphate.*

^2^Seed protein = 6.25 *N concentration (APSIM model uses N concentration).

*^3^PAW, plant available water is defined as the difference between field capacity and wilting point at 0–45 cm depth.*

For climate change scenarios, we updated the baseline weather per location with the projected changes in monthly maximum and minimum temperature and rainfall (Figure 1). Monthly changes were site-specific (Supplementary Table 5). N management scenarios included two fertilization strategies, in the early spring or pod development stage (Mourtzinis et al., 2018), and a high/low initial soil inorganic N to reflect different amounts of leftover N from the previous maize crop, within the ranges reported by Martinez-Feria et al. (2019). Residue management scenarios included alterations in the residue amount (Nunes et al., 2021) and CN ratio (Burgess et al., 2011). We also considered a scenario with a full tillage event before sowing (Daigh et al., 2018). Plant management scenarios included different sowing densities and dates. Sowing densities consisted of 10 plants/m^2^ increase and decrease from baseline, consistent with the ranges reported by Carciochi et al. (2019). We varied the sowing date by ± 12 days from the baseline (50% NASS planting progress) to reflect early and late sowing date, which corresponds to approximately 20 and 80% NASS planting progress by year.

To represent high and low seed protein cultivars we changed the critical seed N concentration thresholds in the APSIM model. Demand for grain N attempts to maintain N at the critical (non-stressed) level (Robertson and Lilley, 2016). For the soil-related scenarios, we altered soil organic carbon (SOC) values across the profile by ± 15%, consistent with the ranges found by Nunes et al. (2020). The changes in SOC were not accompanied by changes in soil water properties (drained upper limit, lower limit, and saturated volumetric water contents). This is justified by the small effect that this level of change in SOC would have on soil water properties and systems outcomes (Palmer et al., 2017). For water management scenarios, we included irrigation and subsurface drainage (Helmers et al., 2012). In the irrigated locations, we added a rainfed scenario (e.g., Mead, NE, United States), while in the rainfed locations we added an irrigation scenario. Similarly, in locations with subsurface drainage (e.g., Nashua, IA, United States), we considered a no subsurface drainage scenario and vice versa. For the irrigation, we used an auto-irrigation rule (see #21 in Table 1) and considered a 5-day interval between irrigations to better represent reality.

### Data Analysis

Data analysis and visualization were conducted in R version 4.1.1 (R Development Core Team, 2010). Data included daily soybean BNF, plant and seed N uptake, net N mineralization, soil nitrate, and N_2_O across a range of scenarios. The coefficient of variation (CV) was calculated across weather years and management scenarios to quantify the contribution of weather and management to the variation of crop and N variables. N balance was calculated as the difference between fixed N in above ground biomass and seed N removal. For the scenarios with N fertilization, this input was accounted in the N balance (BNF + N fertilization–seed N removal). A relative sensitivity index was calculated (Hamby, 1994) to assess the influence of GxExM scenarios on N dynamics.

## Results

### Nitrogen Fluxes Followed Similar Temporal Patterns Across Locations but of Different Magnitude

The temporal patterns in simulated BNF, seed and total aboveground plant N accumulation, soil N mineralization, and nitrate pools were similar among locations, but of different magnitude (Figure 2). Soil nitrate decreased during the growing period, with a sharp evident decline 50 days after sowing (Figure 2). During the seed filling period, soil nitrate was nearly zero with values at crop harvest ranging from 9 to 40 kg N ha^–1^. Cumulative net N mineralization had negative values in the spring reflecting immobilization of inorganic N caused by the maize stover decomposition followed by positive values that were associated with high N mineralization rates during summer. The positive values of mineralization did not increase soil nitrate pool size during the seed filling period because the mineralized N was immediately taken up by the crop. At the end of the season, net N mineralization averaged 73 kg N ha^–1^ with values ranging from 31 kg ha^–1^ (Atkinson, NE, United States) to 121 kg ha^–1^ (Crawfordsville, IA, United States). These two locations had the lowest and highest soil organic matter values (Figure 2).

Biological N fixation accounted on average for 53% of the total aboveground N, with values ranging from 30 to 70% across locations (Figure 2). BNF initiated a week after plant N uptake and ceased at physiological maturity. While BNF was variable across locations, in all cases the cumulative BNF was lower than the seed N accumulation. The seed N accumulation initiated on average 49 days after BNF and followed a much steeper rate of increase (4.8 ± 0.8 kg N ha^–1^ day^–1^) compared to BNF (2.4 ± 0.5 kg N ha^–1^ day^–1^). As a result, the N balance (BNF + N fertilization–seed N removal) had positive values from sowing to about 1/3 of the seed filling period and then declined to negative values at physiological maturity (−21 to −88 kg ha^–1^; Supplementary Figure 2). Even when N balance was calculated by considering more N fluxes such as N loss and N mineralization, the trends were similar (Supplementary Figure 3). Across locations, the simulated 25-year average soybean yield (0% moisture) ranged from 2.1 to 4.3 Mg ha^–1^ (Figure 2).

Cumulative N_2_O fluxes exhibited an east to the west spatial gradient in terms of magnitude with average values ranging from 3.7 kg ha^–1^ year^–1^ in Crawfordsville, IA to 0.2 kg ha^–1^ year^–1^ in Atkinson, NE (Supplementary Figure 4A). Environments with shallow water tables (e.g., Iowa locations) had the highest N_2_O emissions and variance across years. The majority of N_2_O fluxes occurred in the spring (Supplementary Figure 4B).

Grouping the 25-year baseline weather as warm-wet, cool-wet, warm-dry, and cool-dry revealed that the N_2_O emissions had the largest sensitivity to weather patterns when compared to the other N fluxes (Supplementary Figure 5). In general, wet years increased BNF, seed N accumulation, N net mineralization, and N_2_O emissions. Residual soil nitrate at crop maturity was more associated with temperature than rainfall shifts, with lower values under cooler conditions. Site-specific responses were observed (Supplementary Figure 5).

### Equal Contribution of Weather Variability and Management Settings on Nitrogen Fluxes

**FIGURE 3:**
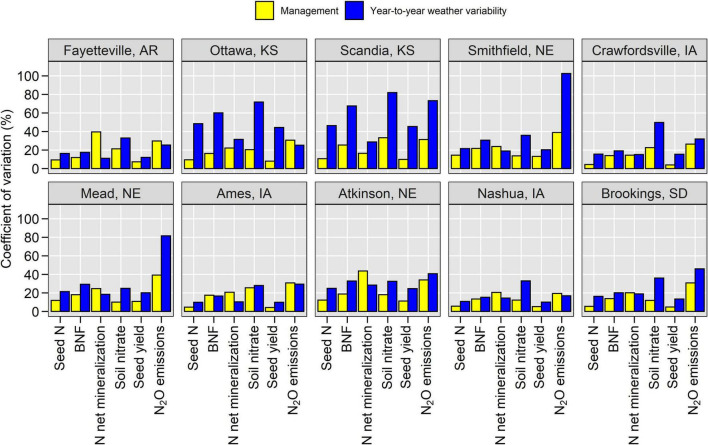
Coefficient of variation for seed N, biological N fixation (BNF), N net mineralization, soil nitrate at harvest, seed yield and N_2_O emissions from different weather years and management scenarios across contrasting environments in the United States Corn Belt.

The year-to-year weather variability accounted for 31% of the variation in N fluxes while management accounted for 32% (average across all locations, Figure 3). The N_2_O flux was influenced the most by weather variability and management (Figure 3). However, N_2_O is also the smallest N flux in terms of magnitude (Supplementary Figure 4). Soil N mineralization and BNF were the next most influenced N fluxes while seed N and yields were the least affected. Hence, soybean yield buffered part of the variability created by management and weather on N fluxes.

Our analysis revealed a strong east to west (wet to dry) spatial gradient on the importance of weather and management (Figure 3). In environments with sufficient-to-excess moisture, management caused more variability in N fluxes than the inter-annual weather variability (e.g., Iowa), while in environments with insufficient moisture, management caused less variability in N fluxes than weather (e.g., Kansas). In irrigated environments (Nebraska and Arkansas), the contribution of weather on N flux variability was slightly lower than management practices. As a result, in environments with water limitations, management-induced changes in N_2_O, BNF, and mineralization fluxes are less likely to be realized.

### GxExM Scenarios Affect Nitrogen Fluxes in Different Ways

**FIGURE 4:**
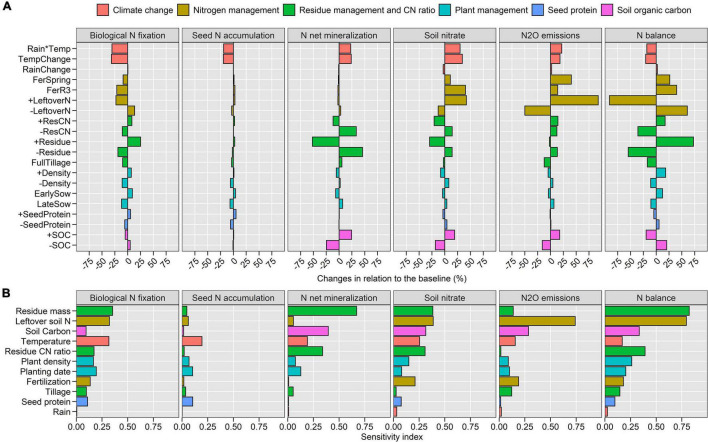
Relative effects of climate change and management scenarios on soil-plant N dynamics compared to the baseline **(A)** and ranking of the importance of different GxExM variables with respect to N fluxes based on a sensitivity analysis **(B)**. Values were averaged over all locations and years. A summary table is also presented in Supplementary Table 6.

**FIGURE 5:**
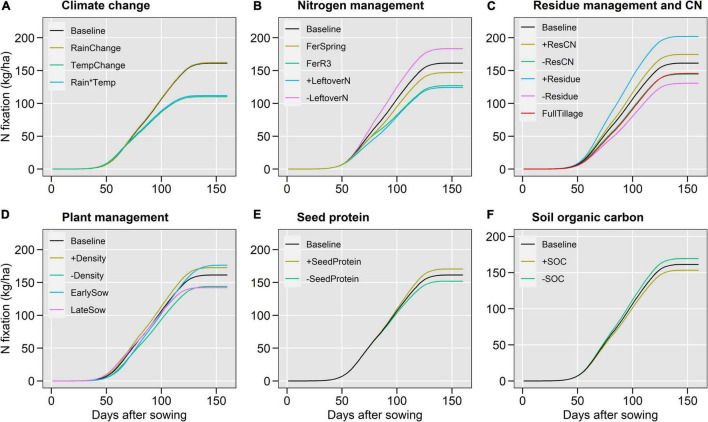
GxExM effects on cumulative aboveground N fixation during the soybean growing season for different scenarios including climate change **(A)**, nitrogen management **(B)**, residue management and quality **(C)**, plant management **(D)**, seed protein **(E)** and soil organic carbon **(F)**. Values were averaged over 10 locations and 25-years per locations.

**FIGURE 6:**
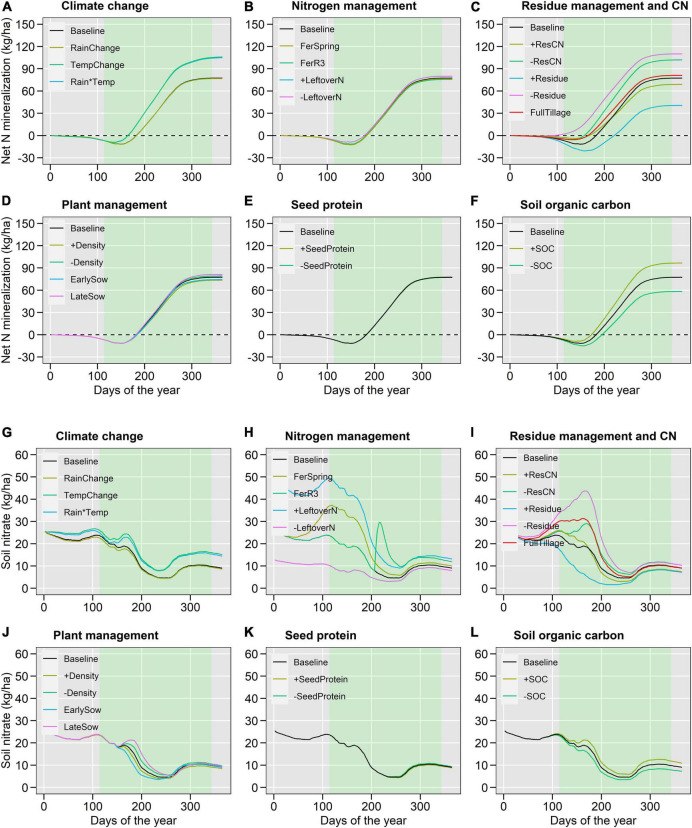
GxExM effects on cumulative net N mineralization **(A–F)** and soil nitrate at 0–30 cm **(G–L)** for different scenarios including climate change **(A,G)**, nitrogen management **(B,H)**, residue management and quality **(C,I)**, plant management **(D,J)**, seed protein **(E,K)** and soil organic carbon **(F,L)**. The shaded area represents the average soybean growing period. Values were averaged over 10 locations and 25-years per locations.

Although specific environments differed in the magnitude of responses to climate and management changes, the general responses were similar and were averaged over all locations to simplify the presentation (Figures 4–6).

#### Climate Change

The 2°C temperature increase under future climate scenarios (Figure 1) decreased BNF by 32% (Figures 4A, 5A), seed N accumulation by 20% (Figure 4A and Supplementary Figure 6A), and N balance by 20% (Figure 4A and Supplementary Figure 7A), while increased N net mineralization by 24% (Figures 4A, 6A), soil nitrate by 34% (Figures 4A, 6G), and N_2_O emissions by 19% (Figure 4A and Supplementary Figure 8A). Changes in rainfall patterns alone had a small effect on N fluxes (up to 3%). The combination of rainfall and temperature changes showed similar responses to single changes in temperature (Figure 4). Seed yield decreased under climate change scenarios, similar to seed N (Supplementary Figure 9A).

#### Nitrogen Management

High soil inorganic N levels created by the application of N fertilizers or carryover nitrate from the previous maize crop (Table 1) decreased BNF up to 23% (Figures 4A, 5B) but increased soil nitrate at crop harvest up to 42% (Figures 4A, 6H) and N_2_O emissions up to 93% (Figure 4A and Supplementary Figure 8B). Seed N accumulation and N net mineralization were minimally affected by N management scenarios (Figures 4, 6B and Supplementary Figure 6B). The application of fertilizers increased the N balance while the high carryover nitrate from the previous cropping years decreased the N balance (Figure 4A and Supplementary Figure 7B).

#### Residue Management and CN Ratio

Crop residue management influenced N fluxes the most (Figure 4B). Large amounts of stover increased BNF by 25% (Figures 4A, 5C), decreased net mineralization by 52%, and soil nitrate at harvest by 30% (Figures 4A, 6C,I). The high CN ratio scenario altered N fluxes similar to the stover scenario but at a threefold lower magnitude. Both large stover amount and high CN ratio increased N balance by 73 and 14%, respectively, because of the increase in BNF. Stover incorporation by tillage decreased 10% BNF, 17% N balance, and 12% N_2_O emissions (Figure 4).

#### Plant Management and Seed Protein

Changes in plant management and seed protein influenced N fluxes considerably less than stover and N management (Figure 4). High plant density, early sowing date, and soybean variety with high seed protein increased BNF by 7, 9, and 6%, respectively (Figures 4, 5). Plant management had little impact on mineralization, seed N, and yield. As a result, the N balance increased (Figure 4).

#### Soil Organic Carbon

A 15% increase in soil organic carbon increased net N mineralization by 25%, soil nitrate by 19%, and N_2_O emissions by 20% (Figures 4A, 6F,L). On the other hand, it decreased BNF by 5% and N balance by 20% (Figures 4A, 5F and Supplementary Figure 7F). Seed N and yield were minimally influenced by increasing soil carbon (<1%; Figure 4 and Supplementary Figures 6F, 9F). After stover and leftover soil N management practices, soil organic carbon showed the greatest influence on N fluxes (Figure 4B).

#### Water Management

To investigate the impact of water source and management (irrigation: water comes from surface vs. shallow water table: water comes from the subsoil) we performed region-specific simulations (Table 2). In the irrigated Nebraska and Arkansas locations, a simulation with no-irrigation revealed that irrigation increased BNF by 58%, seed N by 50%, net mineralization by 66%, and N_2_O emission by 21% while decreasing N balance by 4%. In rainfed Kansas locations, the application of irrigation altered N fluxes similarly to Nebraska and Arkansas (Table 2). However, in rainfed Iowa locations with shallow water tables, irrigation had a substantially lower impact on N fluxes (up to 10%) compared to the impact of irrigation at the eastern locations. In contrast, the presence of shallow water tables in the Iowa locations increased BNF by 21%, seed N by 16%, net mineralization by 10%, and N_2_O emission by 68% while decreasing the N balance by 9%. The subsurface drainage that is used to regulate the water table depth in the Iowa locations had a low impact on BNF and seed N (less than 3%), increased N net mineralization by 9%, and decreased N_2_O emissions and N balance by 24 and 8%, respectively. There were specific years and locations where subsurface drainage was more influential (Supplementary Figure 10).

**TABLE 2:** Simulated impacts of water management on N fluxes (% changes in relation to baseline conditions).

Water management baseline	Scenarios	BNF	Seed N accumulation	N Net Mineralization	Soil nitrate	N_2_O emissions	N balance
Irrigated (Nebraska and Arkansas)	Rainfed	−58	−50	−66	−24	−21	4
Rainfed (Kansas)	Irrigated	43	34	30	−11	7	−27
Rainfed with shallow WT (Ames, Iowa)	Irrigated	−1.5	0.6	8	6	1.1	−4
	No-WT	−25	−20	−12	−11	−72	15
	Tile	−0.5	0.7	3	1.1	−8	−3
Rainfed with shallow WT and tile drainage (Crawfordsville and Nashua, Iowa)	Irrigated	0.7	2	10	9	4	−6
	No-WT	−17	−12	−7	−11	−65	2
	No-Tile	−0.7	−4	−14	−6	41	14

*BNF, biological N fixation; WT, water table.*

## Discussion

### Climate Change Impacts on Nitrogen Fluxes and Soybean Yields

Typically, climate change scenarios combined with uncertain assumptions for the future sowing date, sowing density, and varieties are used to drive crop model simulations to predict seed yield impacts, which generates inherent uncertainties in model predictions (Challinor et al., 2013; Folberth et al., 2016; Corbeels et al., 2018). Here we took a different approach. We performed a sensitivity analysis of both climate change and management settings on whole system processes across 10 soybean production environments to deeper understand and quantify the temporal dynamics of key N fluxes influencing productivity and environmental performance. Therefore, this study provides actionable data for improved management and adaptation to climate change (Thornton et al., 2014; Tui et al., 2021).

Our study revealed three important results: (1) climate change without management adaptation will decrease seed yields and N balance. This is mostly driven by temperature increases rather than shifts in rainfall patterns; (2) climate change will increase mineralization (and thus the soil N pool) and will decrease BNF, therefore, altering the source of N available for plant uptake; and (3) climate change will increase N_2_O emissions because of the larger available mineral N during crop growth.

The decrease in BNF, seed N, and yields were mostly caused by the shortening of the crop growth duration due to increased temperatures (average reduction of 14 days, Supplementary Figure 11), which agrees with other studies (Purcell et al., 2004; Schlenker and Roberts, 2009; Zhang and Cai, 2013; Zipper et al., 2016; Jin et al., 2017; Schauberger et al., 2017; Zhao et al., 2017; Ciampitti et al., 2021). A portion of the anticipated future decline can be offset by adjusting the cultivar maturity group (Kucharik and Serbin, 2008; Zabel et al., 2021). Plant N uptake and BNF are highly coupled with dry matter accumulation (Herridge et al., 2001; Salvagiotti et al., 2008; Santachiara et al., 2017; Córdova et al., 2019), which explains why both N fluxes are decreasing. However, BNF is more sensitive to drought and excess water stress than photosynthesis or mineralization, another aspect that decreases BNF more than other plant–soil processes (Herridge et al., 2001; Purcell et al., 2004; Pasley et al., 2020). This leads to further diminished N balance values at crop harvest (Figure 4 and Supplementary Figure 7). N balance at harvest time had negative values across all assessed scenarios. Santachiara et al. (2017) found that a neutral N balance can be attained when BNF represents 80% of total soybean N uptake. The values found in the present study ranged from 30 to 70% (baseline conditions), which is consistent with the findings of Ciampitti and Salvagiotti (2018).

The increasing N mineralization and topsoil nitrate (Figures 4–6) with the simultaneous decrease in seed N uptake and soybean yields under climate change are of concern. Actions should be taken to manage the unused N after crop harvest, such as using cover crops (Udvardi et al., 2021). Our results revealed a 19% average increase in N_2_O with climate change, which is probably an underestimate because we initialized the model at the start of every year. In the sensitivity analysis, we found that increased amounts of leftover N can increase N_2_O by 93% (Figure 4). These results are supported by Iqbal et al. (2018) who observed increases in N_2_O losses in seasons following dry years (and thus high residual N values).

### Management and Weather Variability Effects on Nitrogen Fluxes

Management practices altered N fluxes at a similar (or larger) magnitude than climate change (Figure 4). This suggests that there are opportunities to improve the productivity and environmental performance of soybean-based systems in the context of a changing climate. We found that N fluxes were affected the most by water management, then by soil management, and finally by plant management (Table 2 and Figure 4). Water management requires long term investment in irrigation infrastructure and is dependent on water availability, while soil and plant management are more feasible and easier to adopt in the short term. Our results suggest that the management of stover (amount and quality) and of the carryover N from the previous year is very important for improving soybean performance in the short term (Figure 4). Large amounts of stover can enhance BNF (in accordance with Xie et al., 2021), decrease soil N pools (as inorganic N is used to break down stover) and reduce N_2_O emissions, which is beneficial. To a smaller extent, plant management such as early sowing date and high plant density could also increase BNF (Figure 4), which is explained by the greater biomass production and yield (Supplementary Figure 9). These findings are consistent with Seneviratne et al. (2000) and de Borja Reis et al. (2021). While not explored in this study, we believe that stacked soil and plant management practices (e.g., Martinez-Feria et al., 2019) can reveal either a higher potential role of management practices to improve soybean-based systems performance and compensate, or even reverse, the negative impacts of climate change on soybean productivity. Future studies could explore this.

Our study revealed the key role of water source (irrigation, water table) and management (subsurface drainage) on N fluxes, which is not surprising given its control over key microbiological processes governing N dynamics. Irrigation had a large influence on N fluxes, increasing BNF, seed N, and mineralization by 50, 42, and 48%, respectively (Table 2). This practice has contributed to high and stable yield levels in the western Corn Belt and is standard practice (Grassini et al., 2014, 2015; Gibson et al., 2018). In the central-east part of the Corn Belt with rainfed crops, the existence of a shallow water table enhanced seed N by 16%, BNF by 21%, and mineralization by 10% compared to a non-water table scenario (Table 2). This result highlights the importance of considering the subsoil moisture on N fluxes, something that has been overlooked in previous studies. The subsurface drainage practice further increased seed N up to 4% (Table 2), which is similar to the findings of Mourtzinis et al. (2021) using experimental data. The small differences in the average results (drained vs. undrained) can be partially explained by the year-to-year weather variability. In dry years, the impact of subsurface drainage on yields and N fluxes was negligible, but in wet years (11 of 25 years; Figure 1) the impact was more pronounced, consistent with Castellano et al. (2019) and Mourtzinis et al. (2021). Subsurface drainage also reduced N_2_O emissions by 24% (Table 2) due to increased soil aeration, which is consistent with Kumar et al. (2014) and Castellano et al. (2019).

Changes in management practices should be evaluated in the context of inter-annual weather variability (Figure 3). We found opportunities to alter N fluxes through management toward decreasing N_2_O and increasing BNF but it is also concerning that an improvement in management can be overwhelmed by weather. An east-to-west gradient on the importance of management vs. weather variability was observed. When water limits crop production, changes in management practices are less likely to alter N fluxes in the desired way (e.g., increase BNF, decrease N_2_O emissions). The CV in N fluxes arising from weather variability was twofold larger than that of management in Kansas (Figure 3). Therefore, management strategies are expected to increase BNF and productivity in regions and years with moderate to no water limitations. These results are supported by Battisti et al. (2018) who found that under favorable climate conditions in Brazil, soybean crop responses to improved management can be maximized. Current findings reinforce the concept that GxExM interactions preclude a single management recommendation for all environments (Serraj et al., 1999; Salvagiotti et al., 2008; Ciampitti and Salvagiotti, 2018). Simulation modeling and machine learning approaches can help in that respect. Future studies could leverage machine learning (e.g., Shahhosseini et al., 2021) to create meta-models using results from process-based models for the identification of relationships between easily measured variables in the field and complex but important processes, such as BNF and N mineralization that are difficult to measure. This could lead to fast assessments and informed decisions.

### The Value of the Systems Approach to Understanding Nitrogen Fluxes in the Context of Climate Change

Our study provides the first systems-level evaluation of soybean N dynamics across a wide range of environments, management practices, and climate change scenarios. This is important for understanding the complex agronomic system and conceptualizing metrics for environmental assessments in soybean-based systems as reported for maize in the United States Corn Belt (Tenorio et al., 2019). The high temporal resolution of generated results aided understanding of when soil N pools and N fluxes -the main determinants of seed N uptake- decrease or increase during the growing season. For example, we learned that seed N accumulation exceeds cumulative BNF around 110 days after sowing, leading to a negative N balance thereafter. If the target is a positive N balance, research should be directed on how to keep seed N below BNF in the last month of soybean growth. The soil nitrate pools declined to near-zero values approximately 50 days after sowing across locations, in accordance with Córdova et al. (2019), which explains the very low N_2_O loss during soybean growth (Supplementary Figure 4). High soil inorganic N levels created by the application of N fertilizers or carryover nitrate from the previous maize crop were the main driver of increased N_2_O emissions, which is consistent with Lu et al. (2021). We found the majority of N_2_O fluxes to occur in the spring, while the magnitude of N_2_O loss followed east to west spatial gradient (from 0.2 to 3.7 kg ha^–1^ year^–1^). We attributed this gradient to higher cumulative rainfall (Figure 1) associated with the existence of shallow water tables in the eastern locations (Table 2).

The process-based systems analysis revealed tradeoffs and synergies among N fluxes (Figures 4–6, e.g., stover effects on BNF and mineralization). We found that the inter-annual weather variability in soybean yield and seed N uptake was less than the variability in other N fluxes (Figure 3). This suggests that soybean buffers are part of the variability caused by management and weather and that there are opportunities to alter N fluxes without compromising yields. Future climate change impact studies should expand the focus from seed yield impacts to whole system evaluation to further understand trade-offs and synergies in different environments.

We acknowledge that the representation of the agronomic system through simulation modeling is a big challenge. No biotic factors were considered in the simulations, which may preclude some identified solutions. While we believe that our modeling approach captures the most important abiotic factors for this study, we recognize that a model will never capture all bio-physical-chemical processes. We also recognize that model structure can bias results (Tao et al., 2018), and using a multi-model approach could increase confidence in the results (Li et al., 2015; Martre et al., 2015; Battisti et al., 2017). In our case, we ensured through extensive testing (Supplementary Figure 1) that the model used here could represent reality well. In fact, very few prior studies have tested a model against so many high-resolution and multi-faceted datasets before its application to answer scientific questions. While the approach we used to evaluate climate change impacts is relatively simple, it gives similar trends compared to more complex approaches such as using daily bias-corrected future climate outputs from RCPs-GCMs combinations (Supplementary Figure 12). Our study also captures important signals of future climate extremes (Supplementary Figure 13). Future studies could explore the impact of a maize-soybean rotation without a yearly reset to quantify potential synergies, negative impacts, and interactions. We also encourage exploring uncertainties in more detail by using daily future climate projections as well as a thorough assessment of climate extremes impacts.

## Conclusion

This study enhances our understanding of the temporal and spatial dynamics of soil-plant N dynamics under current and future climate conditions in the United States. Climate change is expected to increase N mineralization and N_2_O emissions and decrease BNF, seed N, and yields. Management practices altered N fluxes at about the same magnitude as climate change but in many different directions suggesting large opportunities to improve productivity and environmental performance in soybean-based cropping systems. Among many practices explored, we conclude that the management of maize stover (amount and quality) is very important in the short term, and water management (irrigation, subsurface drainage) is critical in the long term. Soybean yield buffered much of the variability caused by management and weather on N fluxes, which creates opportunities to manage N fluxes without compromising yields. This is more likely to be realized in regions with adequate to excess soil moisture.

## Data Availability Statement

The original contributions presented in the study are included in the article/Supplementary Material, further inquiries can be directed to the corresponding author/s.

## Author Contributions

EE contributed to methodology, software, data curation, formal analysis, visualization, and writing – original draft, review, and editing. IC, MC, LP, SN, PG, and PK contributed to fund acquisition, investigation, and writing – review, and editing. NL, LM, and AB contributed to the investigation, writing – review, and editing. SA contributed to the conceptualization, supervision, funding acquisition, methodology, software, and writing – original draft, review, and editing. All authors contributed to the article and approved the submitted version.

## Conflict of Interest

The authors declare that the research was conducted in the absence of any commercial or financial relationships that could be construed as a potential conflict of interest.

## Publisher’s Note

All claims expressed in this article are solely those of the authors and do not necessarily represent those of their affiliated organizations, or those of the publisher, the editors and the reviewers. Any product that may be evaluated in this article, or claim that may be made by its manufacturer, is not guaranteed or endorsed by the publisher.
