# Supplementary material for: Climate Change and Management Impacts on Soybean N Fixation, Soil N Mineralization, N2O Emissions, and Seed Yield
Source: Front Plant Sci. 2022 Apr 27;13:849896. doi: 10.3389/fpls.2022.849896 (PMC9094616; doi:10.3389/fpls.2022.849896)
Supplement: Supplementary file 1 [file Data_Sheet_1.docx]

Supplementary Material

**Climate change and management impacts on soybean N fixation, soil N mineralization, N_2_0 emissions and seed yield**

**Supplementary Table 1.** Experimental field data information

| **Arkansas location (Fayetteville)**  For Arkansas location, experiments were conducted in 2008 and 2009 to evaluate N fixation in a set of near-isolines for maturity in maturity groups 4, 5, and 6 under irrigated conditions (Mastrodomenico & Purcell, 2012). Also included in the experiment was a MG 6 non-nodulating genotype that was a sister line to the maturity isolines (which allowed us to test APSIM’s fixation routine). Field data included time series biomass (total and partitioning), grain yield, N uptake and N fixation. |
| --- |
| **Kansas locations (Scandia and Ottawa)**  For the 2 Kansas rainfed locations, we used experimental data from years 2014-2015 (Scandia) from Balboa et al. (2019) and 2016-2017 (Ottawa) from Hansel (2019). Cultivar maturity groups ranged from 3 to 4.2. Management factors included planting dates, plant densities, and row spacing and fertilization rates. Field data included plant biomass, grain yield and N uptake. |
| **Nebraska locations (Mead, Atkinson and Smithfield)**  For the 3 NE locations, we used data from high-yielding irrigation experiments conducted from 2015 to 2017 (Cafaro La Menza et al., 2017, 2019, 2020). Cultivar maturity groups ranged from 2.4 to 2.7. Management included full-N and zero-N fertilization. Field data included time series biomass (total and partitioning), grain yield, N uptake and N fixation. |
| **Iowa locations (Ames, Crawfordsville and Nashua)**  For the 3 rainfed Iowa locations, we used data from Archontoulis et al. (2020) and (Córdova et al., 2019). The data included time series on crop biomass, grain yield, N uptake, soil nitrate by layer and N fixation. Management factors included planting dates (2015-2017) and high vs low input management (2018-2019) in Ames. In Crawfordsville, the factors were subsurface drainage systems vs no drainage (2016-2018). In Nashua, there was no management factors (2016-2018). Cultivar maturity groups ranged from 2.2 to 3.2. |
| **South Dakota location (Brookings)**  For the south Dakota location, we used data from experiments conducted from 2018 to 2019 (unpublished data) using cultivar maturity groups 1 and 2. The dataset included time series biomass (total and partitioning) and grain yield. |

**References for further information on experimental field data**

Archontoulis, S. V., Castellano, M. J., Licht, M. A., Nichols, V., Baum, M., Huber, I., Martinez‐Feria, R., Puntel, L., Ordóñez, R. A., Iqbal, J., Wright, E. E., Dietzel, R. N., Helmers, M., Vanloocke, A., Liebman, M., Hatfield, J. L., Herzmann, D., Córdova, S. C., Edmonds, P., … Lamkey, K. R. (2020). Predicting crop yields and soil‐plant nitrogen dynamics in the US Corn Belt. Crop Science, 60(2), 721–738. doi: 10.1002/csc2.20039

Balboa, G. R., Archontoulis, S. V., Salvagiotti, F., Garcia, F. O., Stewart, W. M., Francisco, E., Prasad, P. V. V., & Ciampitti, I. A. (2019). A systems-level yield gap assessment of maize-soybean rotation under high- and low-management inputs in the Western US Corn Belt using APSIM. Agricultural Systems, 174, 145–154. doi: 10.1016/j.agsy.2019.04.008

Cafaro La Menza, N., Monzon, J. P., Lindquist, J. L., Arkebauer, T. J., Knops, J. M. H., Unkovich, M., Specht, J. E., & Grassini, P. (2020). Insufficient nitrogen supply from symbiotic fixation reduces seasonal crop growth and nitrogen mobilization to seed in highly productive soybean crops. Plant, Cell & Environment, 43(8), 1958–1972. doi: 10.1111/PCE.13804

Cafaro La Menza, N., Monzon, J. P., Specht, J. E., & Grassini, P. (2017). Is soybean yield limited by nitrogen supply? Field Crops Research, 213, 204–212. doi: 10.1016/J.FCR.2017.08.009

Cafaro La Menza, N., Monzon, J. P., Specht, J. E., Lindquist, J. L., Arkebauer, T. J., Graef, G., & Grassini, P. (2019). Nitrogen limitation in high-yield soybean: Seed yield, N accumulation, and N-use efficiency. Field Crops Research, 237, 74–81. doi: 10.1016/J.FCR.2019.04.009

Córdova, S. C., Castellano, M. J., Dietzel, R., Licht, M. A., Togliatti, K., Martinez-Feria, R., & Archontoulis, S. V. (2019). Soybean nitrogen fixation dynamics in Iowa, USA. Field Crops Research, 236, 165–176. doi: 10.1016/j.fcr.2019.03.018

Hansel, D. S. S. (2019). Double crop soybeans management: a review, field studies, and modeling. PhD Thesis. Kansas State University. 102 p.

Mastrodomenico, A. T., & Purcell, L. C. (2012). Soybean Nitrogen Fixation and Nitrogen Remobilization during Reproductive Development. Crop Science, 52(3), 1281–1289. doi: 10.2135/CROPSCI2011.08.0414

**Supplementary Table 2.** Calibrated Soybean cultivar parameters used in APSIM.

| Location | Cultivar MG | Cultivar parameters | Units | Value/Array |
| --- | --- | --- | --- | --- |
| Fayett., AR | 4.0 | node_sen_rate | oCd/node | 120 |
|  |  | x_pp | H | 13.09, 13.9, 14.8, 15.6 |
|  |  | y_tt_end_of_juvenile | oCd | 100, 133, 200, 400 |
|  |  | y_tt_floral_initiation | oCd | 190, 253, 380, 760 |
|  |  | y_tt_flowering | oCd | 263, 351, 527, 1404 |
|  |  | y_tt_start_grain_fill | oCd | 535, 713, 1069, 2852 |
| Fayett., AR | 5.0 | node_sen_rate | oCd/node | 120 |
|  |  | x_pp | H | 12.83, 13.7, 14.5, 15.3 |
|  |  | y_tt_end_of_juvenile | oCd | 100, 133, 200, 400 |
|  |  | y_tt_floral_initiation | oCd | 230, 307, 460, 920 |
|  |  | y_tt_flowering | oCd | 271, 361, 541, 1443 |
|  |  | y_tt_start_grain_fill | oCd | 549, 732, 1099, 2929 |
| Fayett., AR | 6.0 | node_sen_rate | oCd/node | 120 |
|  |  | x_pp | H | 12.58, 13.4, 14.2, 15.0 |
|  |  | y_tt_end_of_juvenile | oCd | 100, 133, 200, 400 |
|  |  | y_tt_floral_initiation | oCd | 250, 333, 500, 1000 |
|  |  | y_tt_flowering | oCd | 278, 370, 555, 1481 |
|  |  | y_tt_start_grain_fill | oCd | 564, 752, 1128, 3007 |
| Ottawa, KS | 4.2 | node_sen_rate | oCd/node | 140 |
|  |  | x_pp | H | 13.09, 13.9, 14.8, 15.6 |
|  |  | y_tt_end_of_juvenile | oCd | 200, 233, 200, 400 |
|  |  | y_tt_floral_initiation | oCd | 192, 223, 185, 370 |
|  |  | y_tt_flowering | oCd | 363, 451, 527, 1404 |
|  |  | y_tt_start_grain_fill | oCd | 535, 713, 1069, 2930 |
| Scandia, KS | 3.0 | node_sen_rate | oCd/node | 120 |
|  |  | x_pp | H | 13.4, 14.3, 15.2, 16.0 |
|  |  | y_tt_end_of_juvenile | oCd | 100, 133, 200, 400 |
|  |  | y_tt_floral_initiation | oCd | 160, 213, 320, 640 |
|  |  | y_tt_flowering | oCd | 253, 338, 506, 1350 |
|  |  | y_tt_start_grain_fill | oCd | 514, 685, 1028, 2741 |
| Smith., NE | 2.4 | node_sen_rate | oCd/node | 120 |
|  |  | x_pp | H | 13.4, 14.3, 15.2, 16.0 |
|  |  | y_tt_end_of_juvenile | oCd | 100, 133, 200, 400 |
|  |  | y_tt_floral_initiation | oCd | 130, 213, 320, 640 |
|  |  | y_tt_flowering | oCd | 290, 400, 600, 1759 |
|  |  | y_tt_start_grain_fill | oCd | 437, 583, 875, 2332 |
| Crawf., IA | 3.2 | node_sen_rate | oCd/node | 120 |
|  |  | x_pp | H | 13.4, 14.3, 15.2, 16.0 |
|  |  | y_tt_end_of_juvenile | oCd | 100, 133, 200, 400 |
|  |  | y_tt_floral_initiation | oCd | 160, 213, 320, 640 |
|  |  | y_tt_flowering | oCd | 253, 338, 506, 1350 |
|  |  | y_tt_start_grain_fill | oCd | 440, 580, 1028, 2741 |
| Mead, NE | 2.7 | node_sen_rate | oCd/node | 120 |
|  |  | x_pp | H | 13.4, 14.3, 15.2, 16.0 |
|  |  | y_tt_end_of_juvenile | oCd | 100, 133, 200, 400 |
|  |  | y_tt_floral_initiation | oCd | 120, 180, 260, 640 |
|  |  | y_tt_flowering | oCd | 250, 350, 550, 1759 |
|  |  | y_tt_start_grain_fill | oCd | 497, 583, 875, 2332 |
| Ames, IA | 2.7 | node_sen_rate | oCd/node | 115 |
|  |  | x_pp | H | 13.5, 14.5, 15.4, 16.3 |
|  |  | y_tt_end_of_juvenile | oCd | 100, 133, 200, 400 |
|  |  | y_tt_floral_initiation | oCd | 128, 171, 256, 512 |
|  |  | y_tt_flowering | oCd | 246, 328, 492, 1312 |
|  |  | y_tt_start_grain_fill | oCd | 499, 666, 999, 2664 |
| Atkin., NE | 2.7 | node_sen_rate | oCd/node | 120 |
|  |  | x_pp | H | 13.4, 14.3, 15.2, 16.0 |
|  |  | y_tt_end_of_juvenile | oCd | 100, 133, 200, 400 |
|  |  | y_tt_floral_initiation | oCd | 120, 180, 260, 640 |
|  |  | y_tt_flowering | oCd | 250, 350, 550, 1759 |
|  |  | y_tt_start_grain_fill | oCd | 497, 583, 875, 2332 |
| Nashua, IA | 2.2 | node_sen_rate | oCd/node | 115 |
|  |  | x_pp | H | 13.5, 14.5, 15.4, 16.3 |
|  |  | y_tt_end_of_juvenile | oCd | 100, 133, 200, 400 |
|  |  | y_tt_floral_initiation | oCd | 128, 171, 256, 512 |
|  |  | y_tt_flowering | oCd | 246, 328, 492, 1312 |
|  |  | y_tt_start_grain_fill | oCd | 499, 666, 999, 2664 |
| Brook., SD | 1.0 | node_sen_rate | oCd/node | 120 |
|  |  | x_pp | H | 13.84, 15.1, 16.3, 17.5 |
|  |  | y_tt_end_of_juvenile | oCd | 100, 133, 200, 400 |
|  |  | y_tt_floral_initiation | oCd | 120, 160, 240, 480 |
|  |  | y_tt_flowering | oCd | 239, 318, 478, 1274 |
|  |  | y_tt_start_grain_fill | oCd | 485, 646, 970, 2586 |
| Brook., SD | 2.0 | node_sen_rate | oCd/node | 120 |
|  |  | x_pp | H | 13.59, 14.6, 15.6, 16.6 |
|  |  | y_tt_end_of_juvenile | oCd | 100, 133, 200, 400 |
|  |  | y_tt_floral_initiation | oCd | 128, 171, 256, 512 |
|  |  | y_tt_flowering | oCd | 246, 328, 492, 1312 |
|  |  | y_tt_start_grain_fill | oCd | 499, 666, 999, 2664 |

**Supplementary Table 3.** Hydrological parameters, soil organic matter and initial nitrogen conditions for the 10 locations assessed in the present study. BD = bulk density, LL15 = lower limit, DUL = drained upper limit, SAT = saturation, OC = soil organic carbon, Fbiom = fraction of OC to BIOM pool, Finert = fraction of OC to INERT pool, NO3 = soil nitrate, NH4 = soil ammonium.

| Location | Depth | BD | LL15 | DUL | SAT | OC | Fbiom | Finert | NO3 | NH4 |
| --- | --- | --- | --- | --- | --- | --- | --- | --- | --- | --- |
|  | cm | g/cm^3^ | mm/mm | | | % | 0-1 | 0-1 | Kg/ha | Kg/ha |
| Fayett., AR | 0-30 | 1.42 | 0.11 | 0.31 | 0.43 | 1.06 | 0.07 | 0.44 | 25.71 | 25.71 |
|  | 30-60 | 1.66 | 0.16 | 0.35 | 0.45 | 0.40 | 0.03 | 0.71 | 16.87 | 16.87 |
|  | 60-90 | 1.64 | 0.18 | 0.36 | 0.45 | 0.24 | 0.02 | 0.75 | 3.91 | 3.91 |
|  | 90-120 | 1.64 | 0.18 | 0.36 | 0.45 | 0.16 | 0.02 | 0.90 | 3.91 | 3.91 |
|  | 120-150 | 1.64 | 0.18 | 0.36 | 0.45 | 0.13 | 0.02 | 0.98 | 3.35 | 3.35 |
|  | 150-180 | 1.64 | 0.18 | 0.36 | 0.45 | 0.06 | 0.01 | 1.00 | 2.76 | 2.76 |
|  | 180-210 | 1.64 | 0.18 | 0.36 | 0.45 | 0.02 | 0.01 | 1.00 | 2.67 | 2.67 |
|  | 210-240 | 1.64 | 0.18 | 0.36 | 0.45 | 0.01 | 0.01 | 1.00 | 2.67 | 2.67 |
|  | 240-270 | 1.64 | 0.18 | 0.36 | 0.45 | 0.00 | 0.01 | 1.00 | 2.67 | 2.67 |
|  | 270-300 | 1.64 | 0.18 | 0.36 | 0.45 | 0.00 | 0.01 | 1.00 | 2.67 | 2.67 |
| Ottawa, KS | 0-30 | 1.32 | 0.17 | 0.30 | 0.50 | 2.33 | 0.05 | 0.40 | 25.71 | 25.71 |
|  | 30-60 | 1.40 | 0.25 | 0.32 | 0.47 | 1.50 | 0.03 | 0.73 | 16.87 | 16.87 |
|  | 60-90 | 1.40 | 0.25 | 0.32 | 0.47 | 1.33 | 0.02 | 0.90 | 3.91 | 3.91 |
|  | 90-120 | 1.40 | 0.24 | 0.31 | 0.47 | 0.83 | 0.02 | 0.97 | 3.91 | 3.91 |
|  | 120-150 | 1.40 | 0.22 | 0.30 | 0.47 | 0.50 | 0.02 | 1.00 | 3.35 | 3.35 |
|  | 150-180 | 1.40 | 0.22 | 0.30 | 0.39 | 0.50 | 0.01 | 1.00 | 2.76 | 2.76 |
|  | 180-210 | 1.40 | 0.22 | 0.30 | 0.38 | 0.50 | 0.01 | 1.00 | 2.67 | 2.67 |
|  | 210-240 | 1.40 | 0.22 | 0.30 | 0.37 | 0.50 | 0.01 | 1.00 | 2.67 | 2.67 |
|  | 240-270 | 1.40 | 0.22 | 0.30 | 0.36 | 0.50 | 0.01 | 1.00 | 2.67 | 2.67 |
|  | 270-300 | 1.40 | 0.22 | 0.30 | 0.36 | 0.50 | 0.01 | 1.00 | 2.67 | 2.67 |
| Scandia, KS | 0-30 | 1.37 | 0.12 | 0.28 | 0.46 | 1.67 | 0.07 | 0.45 | 25.71 | 25.71 |
|  | 30-60 | 1.35 | 0.17 | 0.31 | 0.47 | 1.12 | 0.03 | 0.78 | 16.87 | 16.87 |
|  | 60-90 | 1.37 | 0.18 | 0.29 | 0.46 | 0.41 | 0.02 | 0.87 | 3.91 | 3.91 |
|  | 90-120 | 1.33 | 0.15 | 0.29 | 0.47 | 0.21 | 0.01 | 0.97 | 3.91 | 3.91 |
|  | 120-150 | 1.33 | 0.15 | 0.29 | 0.47 | 0.17 | 0.01 | 0.99 | 3.35 | 3.35 |
|  | 150-180 | 1.33 | 0.15 | 0.29 | 0.47 | 0.10 | 0.01 | 0.99 | 2.76 | 2.76 |
|  | 180-210 | 1.33 | 0.15 | 0.29 | 0.47 | 0.10 | 0.01 | 0.99 | 2.67 | 2.67 |
|  | 210-240 | 1.33 | 0.15 | 0.29 | 0.47 | 0.10 | 0.01 | 0.99 | 2.67 | 2.67 |
|  | 240-270 | 1.33 | 0.15 | 0.29 | 0.47 | 0.10 | 0.01 | 0.99 | 2.67 | 2.67 |
|  | 270-300 | 1.33 | 0.15 | 0.29 | 0.47 | 0.10 | 0.01 | 0.99 | 2.67 | 2.67 |
| Smith., NE | 0-30 | 1.38 | 0.13 | 0.35 | 0.46 | 1.91 | 0.09 | 0.40 | 25.71 | 25.71 |
|  | 30-60 | 1.37 | 0.19 | 0.35 | 0.45 | 1.51 | 0.03 | 0.75 | 16.87 | 16.87 |
|  | 60-90 | 1.33 | 0.14 | 0.33 | 0.46 | 1.13 | 0.02 | 0.83 | 3.91 | 3.91 |
|  | 90-120 | 1.30 | 0.11 | 0.27 | 0.48 | 0.46 | 0.02 | 0.96 | 3.91 | 3.91 |
|  | 120-150 | 1.30 | 0.10 | 0.26 | 0.48 | 0.20 | 0.02 | 0.98 | 3.35 | 3.35 |
|  | 150-180 | 1.30 | 0.10 | 0.26 | 0.42 | 0.20 | 0.01 | 1.00 | 2.76 | 2.76 |
|  | 180-210 | 1.30 | 0.10 | 0.26 | 0.41 | 0.20 | 0.01 | 1.00 | 2.67 | 2.67 |
|  | 210-240 | 1.30 | 0.10 | 0.26 | 0.41 | 0.20 | 0.01 | 1.00 | 2.67 | 2.67 |
|  | 240-270 | 1.30 | 0.10 | 0.26 | 0.40 | 0.20 | 0.01 | 1.00 | 2.67 | 2.67 |
|  | 270-300 | 1.30 | 0.10 | 0.26 | 0.39 | 0.20 | 0.01 | 1.00 | 2.67 | 2.67 |
| Crawf., IA | 0-30 | 1.26 | 0.12 | 0.35 | 0.52 | 2.70 | 0.10 | 0.40 | 25.70 | 25.70 |
|  | 30-60 | 1.34 | 0.18 | 0.35 | 0.49 | 1.48 | 0.03 | 0.63 | 16.92 | 16.92 |
|  | 60-90 | 1.42 | 0.21 | 0.38 | 0.48 | 0.23 | 0.02 | 0.82 | 3.90 | 3.90 |
|  | 90-120 | 1.44 | 0.21 | 0.38 | 0.48 | 0.23 | 0.02 | 0.85 | 3.90 | 3.90 |
|  | 120-150 | 1.46 | 0.20 | 0.37 | 0.46 | 0.23 | 0.02 | 1.00 | 3.35 | 3.35 |
|  | 150-180 | 1.46 | 0.20 | 0.37 | 0.42 | 0.23 | 0.01 | 1.00 | 2.75 | 2.75 |
|  | 180-210 | 1.46 | 0.20 | 0.37 | 0.42 | 0.23 | 0.01 | 1.00 | 2.67 | 2.67 |
|  | 210-240 | 1.47 | 0.20 | 0.37 | 0.42 | 0.23 | 0.01 | 1.00 | 2.67 | 2.67 |
|  | 240-270 | 1.60 | 0.20 | 0.37 | 0.41 | 0.23 | 0.01 | 1.00 | 2.67 | 2.67 |
|  | 270-300 | 1.60 | 0.20 | 0.37 | 0.41 | 0.23 | 0.01 | 1.00 | 2.67 | 2.67 |
| Mead, NE | 0-30 | 1.37 | 0.14 | 0.39 | 0.44 | 1.82 | 0.08 | 0.44 | 25.71 | 25.71 |
|  | 30-60 | 1.32 | 0.18 | 0.37 | 0.42 | 1.24 | 0.03 | 0.73 | 16.87 | 16.87 |
|  | 60-90 | 1.30 | 0.20 | 0.36 | 0.43 | 0.98 | 0.02 | 0.83 | 3.91 | 3.91 |
|  | 90-120 | 1.30 | 0.18 | 0.32 | 0.43 | 0.87 | 0.02 | 0.92 | 3.91 | 3.91 |
|  | 120-150 | 1.31 | 0.17 | 0.30 | 0.42 | 0.25 | 0.02 | 0.97 | 3.35 | 3.35 |
|  | 150-180 | 1.32 | 0.15 | 0.29 | 0.41 | 0.20 | 0.01 | 1.00 | 2.76 | 2.76 |
|  | 180-210 | 1.32 | 0.15 | 0.29 | 0.40 | 0.20 | 0.01 | 1.00 | 2.67 | 2.67 |
|  | 210-240 | 1.32 | 0.15 | 0.29 | 0.40 | 0.20 | 0.01 | 1.00 | 2.67 | 2.67 |
|  | 240-270 | 1.32 | 0.15 | 0.29 | 0.39 | 0.20 | 0.01 | 1.00 | 2.67 | 2.67 |
|  | 270-300 | 1.32 | 0.15 | 0.29 | 0.38 | 0.20 | 0.01 | 1.00 | 2.67 | 2.67 |
| Ames, IA | 0-30 | 1.25 | 0.16 | 0.28 | 0.42 | 2.33 | 0.11 | 0.45 | 25.70 | 25.70 |
|  | 30-60 | 1.35 | 0.11 | 0.22 | 0.34 | 1.46 | 0.03 | 0.81 | 16.87 | 16.87 |
|  | 60-90 | 1.36 | 0.13 | 0.26 | 0.39 | 0.73 | 0.02 | 0.86 | 3.90 | 3.90 |
|  | 90-120 | 1.48 | 0.13 | 0.23 | 0.39 | 0.49 | 0.02 | 1.00 | 3.90 | 3.90 |
|  | 120-150 | 1.60 | 0.09 | 0.20 | 0.39 | 0.49 | 0.02 | 1.00 | 3.35 | 3.35 |
|  | 150-180 | 1.60 | 0.09 | 0.20 | 0.29 | 0.49 | 0.01 | 1.00 | 2.75 | 2.75 |
|  | 180-210 | 1.60 | 0.09 | 0.20 | 0.27 | 0.49 | 0.01 | 1.00 | 2.67 | 2.67 |
|  | 210-240 | 1.60 | 0.09 | 0.20 | 0.26 | 0.49 | 0.01 | 1.00 | 2.67 | 2.67 |
|  | 240-270 | 1.60 | 0.09 | 0.20 | 0.25 | 0.10 | 0.01 | 1.00 | 2.67 | 2.67 |
|  | 270-300 | 1.60 | 0.09 | 0.20 | 0.25 | 0.10 | 0.01 | 1.00 | 2.67 | 2.67 |
| Atkin., NE | 0-30 | 1.52 | 0.09 | 0.24 | 0.43 | 1.32 | 0.06 | 0.37 | 25.71 | 25.71 |
|  | 30-60 | 1.73 | 0.04 | 0.11 | 0.34 | 0.62 | 0.03 | 0.74 | 16.87 | 16.87 |
|  | 60-90 | 1.77 | 0.03 | 0.10 | 0.33 | 0.27 | 0.02 | 0.86 | 3.91 | 3.91 |
|  | 90-120 | 1.77 | 0.03 | 0.10 | 0.33 | 0.18 | 0.02 | 0.88 | 3.91 | 3.91 |
|  | 120-150 | 1.61 | 0.05 | 0.11 | 0.33 | 0.17 | 0.02 | 0.90 | 3.35 | 3.35 |
|  | 150-180 | 1.61 | 0.05 | 0.11 | 0.26 | 0.17 | 0.01 | 0.91 | 2.76 | 2.76 |
|  | 180-210 | 1.61 | 0.05 | 0.11 | 0.25 | 0.17 | 0.01 | 0.93 | 2.67 | 2.67 |
|  | 210-240 | 1.61 | 0.05 | 0.11 | 0.24 | 0.17 | 0.01 | 0.94 | 2.67 | 2.67 |
|  | 240-270 | 1.61 | 0.05 | 0.11 | 0.23 | 0.17 | 0.01 | 0.96 | 2.67 | 2.67 |
|  | 270-300 | 1.61 | 0.05 | 0.11 | 0.22 | 0.17 | 0.01 | 0.97 | 2.67 | 2.67 |
| Nashua, IA | 0-30 | 1.25 | 0.10 | 0.26 | 0.41 | 2.35 | 0.13 | 0.39 | 25.70 | 25.70 |
|  | 30-60 | 1.35 | 0.11 | 0.24 | 0.38 | 1.39 | 0.03 | 0.71 | 16.87 | 16.87 |
|  | 60-90 | 1.56 | 0.12 | 0.25 | 0.39 | 0.56 | 0.02 | 0.75 | 3.91 | 3.91 |
|  | 90-120 | 1.56 | 0.14 | 0.27 | 0.38 | 0.34 | 0.02 | 0.78 | 3.91 | 3.91 |
|  | 120-150 | 1.73 | 0.13 | 0.27 | 0.38 | 0.23 | 0.02 | 0.83 | 3.35 | 3.35 |
|  | 150-180 | 1.82 | 0.14 | 0.27 | 0.32 | 0.13 | 0.01 | 1.00 | 2.76 | 2.76 |
|  | 180-210 | 1.84 | 0.15 | 0.27 | 0.32 | 0.10 | 0.01 | 1.00 | 2.67 | 2.67 |
|  | 210-240 | 1.84 | 0.14 | 0.27 | 0.31 | 0.12 | 0.01 | 1.00 | 2.67 | 2.67 |
|  | 240-270 | 1.84 | 0.13 | 0.27 | 0.30 | 0.12 | 0.01 | 1.00 | 2.67 | 2.67 |
|  | 270-300 | 1.84 | 0.15 | 0.27 | 0.30 | 0.10 | 0.01 | 1.00 | 2.67 | 2.67 |
| Brook., SD | 0-30 | 1.31 | 0.18 | 0.32 | 0.51 | 2.59 | 0.07 | 0.44 | 25.71 | 25.71 |
|  | 30-60 | 1.43 | 0.17 | 0.31 | 0.46 | 2.11 | 0.03 | 0.71 | 16.87 | 16.87 |
|  | 60-90 | 1.47 | 0.16 | 0.30 | 0.44 | 1.74 | 0.02 | 0.75 | 3.91 | 3.91 |
|  | 90-120 | 1.47 | 0.16 | 0.30 | 0.44 | 1.74 | 0.02 | 0.90 | 3.91 | 3.91 |
|  | 120-150 | 1.52 | 0.14 | 0.28 | 0.43 | 1.09 | 0.02 | 0.98 | 3.35 | 3.35 |
|  | 150-180 | 1.53 | 0.16 | 0.32 | 0.42 | 0.17 | 0.01 | 1.00 | 2.76 | 2.76 |
|  | 180-210 | 1.50 | 0.18 | 0.34 | 0.38 | 0.04 | 0.01 | 1.00 | 2.67 | 2.67 |
|  | 210-240 | 1.50 | 0.18 | 0.34 | 0.38 | 0.01 | 0.01 | 1.00 | 2.67 | 2.67 |
|  | 240-270 | 1.50 | 0.18 | 0.34 | 0.38 | 0.00 | 0.01 | 1.00 | 2.67 | 2.67 |
|  | 270-300 | 1.50 | 0.18 | 0.34 | 0.37 | 0.00 | 0.01 | 1.00 | 2.67 | 2.67 |


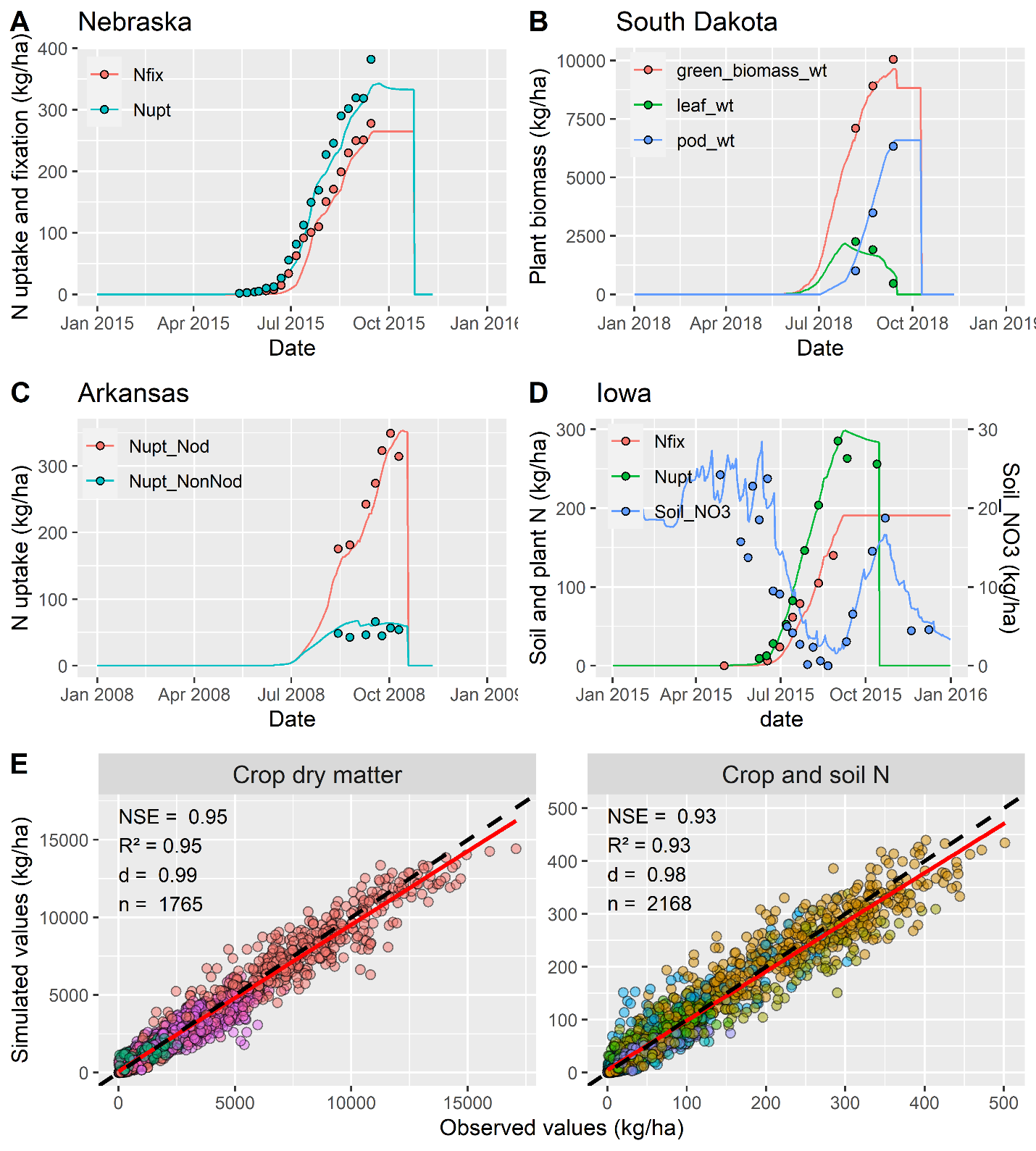


**Supplementary Figure 1.** Measured versus simulated soybean growth and N dynamics across contrasting environmental and management conditions in the US Corn Belt. Panel “A” shows nitrogen fixation and uptake in Atkinson, NE; panel “B” demonstrates total aboveground biomass, leaf biomass and pod biomass in Brookings, South Dakota; panel “C” shows N uptake under conditions with and without biological N fixation in Fayetteville, AR; panel “D” represents Nfix, Nupt and topsoil (0-30 cm) nitrate in Ames, IA. Panel “E” demonstrates the relationship between measured and simulated values considering all field data (including crop dry matter and N variables) used for APSIM model evaluation, in which NSE is the Modelling Efficiency Index (Nash and Sutcliffe, 1970), d the Willmott Agreement Index (Willmott, 1981), R^2^ the coefficient of determination and n the number of data. Colored circles represent different crop and N variables.

**APSIM model performance**

In Supplementary Figure 1 we show an example of day-by-day N uptake and fixation simulations for one Nebraska location, biomass partitioning in South Dakota, N uptake from N fixing and no N fixing cultivars in Arkansas, as well as N uptake, fixation and soil nitrate in Iowa. The overall model performance was adequate, with a Modeling Efficiency Index of 0.95 for crop dry matter variables and 0.93 for N dynamics variables. The model showed also sufficient precision (R^2^ = 0.95 and 0.93 for crop dry matter and N variables, respectively). More detailed information is presented in Supplementary Table 4.

**References for statistical indices (Supplementary Figure 2)**

Nash, J.E., Sutcliffe, J.V., 1970. River flow forecasting through conceptual models part I - A discussion of principles. J. Hydrol. 10, 282–290. doi: 10.1016/0022-1694(70)90255-6

Willmott, C.J., 1981. On the validation of models. Phys. Geogr. 2, 184–194.

**Supplementary Table 4.** Statistical indices from the relationship between measured and simulated values of Crop and N variables across various environments in the US Corn Belt. NSE = Modelling Efficiency Index, d = Willmott Agreement Index, R^2^ = coefficient of determination and n = number of data.

| Data type | Variable | NSE | d | R^2^ | n |
| --- | --- | --- | --- | --- | --- |
|  | Above ground biomass | 0.95 | 0.99 | 0.95 | 670 |
|  | Leaf biomass | 0.78 | 0.94 | 0.79 | 425 |
|  | Stem biomass | 0.83 | 0.95 | 0.84 | 439 |
|  | Grain yield | 0.85 | 0.96 | 0.86 | 231 |
| Crop and soil N | Total N uptake | 0.93 | 0.98 | 0.93 | 622 |
|  | Leaf N uptake | 0.81 | 0.95 | 0.83 | 392 |
|  | Stem N uptake | 0.78 | 0.93 | 0.81 | 396 |
|  | Grain N uptake | 0.84 | 0.95 | 0.88 | 177 |
|  | N fixation | 0.81 | 0.95 | 0.83 | 203 |
|  | Soil NO_3_ (0-30 cm) | 0.67 | 0.89 | 0.68 | 378 |

**Supplementary Table 5.** Monthly changes for future maximum and minimum temperature and rainfall in relation to the baseline for the 10 locations assessed in the present study.

| Location | Month | Maximum temperature (°C) | Minimum temperature (°C) | Rainfall (%) |
| --- | --- | --- | --- | --- |
| Fayetteville, AR | Jan | 2.0 | 1.9 | 5.2 |
|  | Feb | 1.4 | 1.1 | 11.0 |
|  | Mar | 1.5 | 1.3 | 5.0 |
|  | Apr | 1.8 | 1.8 | 9.5 |
|  | May | 2.2 | 1.9 | 6.7 |
|  | Jun | 2.3 | 2.0 | -4.3 |
|  | Jul | 2.0 | 1.9 | 7.5 |
|  | Aug | 2.1 | 1.9 | 5.6 |
|  | Sep | 2.6 | 2.3 | -6.5 |
|  | Oct | 2.6 | 2.3 | -4.4 |
|  | Nov | 2.1 | 1.7 | 5.6 |
|  | Dec | 1.8 | 1.8 | 8.9 |
| Ottawa, KS | Jan | 2.1 | 2.1 | 6.7 |
|  | Feb | 1.3 | 1.1 | 4.3 |
|  | Mar | 1.6 | 1.4 | 2.8 |
|  | Apr | 1.9 | 1.8 | 10.4 |
|  | May | 2.2 | 1.8 | 6.9 |
|  | Jun | 2.3 | 2.1 | 1.3 |
|  | Jul | 2.1 | 1.9 | -0.5 |
|  | Aug | 2.3 | 2.0 | -8.6 |
|  | Sep | 2.7 | 2.4 | -12.2 |
|  | Oct | 2.8 | 2.4 | 6.1 |
|  | Nov | 2.1 | 1.8 | 7.2 |
|  | Dec | 1.9 | 2.2 | 6.9 |
| Scandia, KS | Jan | 1.9 | 1.8 | 7.6 |
|  | Feb | 1.2 | 1.1 | 2.7 |
|  | Mar | 1.6 | 1.5 | 4.9 |
|  | Apr | 1.9 | 1.8 | 7.6 |
|  | May | 2.1 | 1.8 | 8.4 |
|  | Jun | 2.3 | 2.1 | -0.1 |
|  | Jul | 1.9 | 1.9 | -2.1 |
|  | Aug | 2.3 | 2.1 | -13.5 |
|  | Sep | 2.6 | 2.5 | -0.5 |
|  | Oct | 2.9 | 2.5 | -2.1 |
|  | Nov | 2.0 | 1.8 | 24.8 |
|  | Dec | 1.8 | 2.1 | 20.5 |
| Smithfield, NE | Jan | 1.6 | 1.8 | 17.2 |
|  | Feb | 1.1 | 1.1 | 7.7 |
|  | Mar | 1.6 | 1.5 | 9.5 |
|  | Apr | 1.9 | 1.8 | 11.5 |
|  | May | 2.1 | 1.8 | 2.3 |
|  | Jun | 2.5 | 2.0 | -2.7 |
|  | Jul | 2.1 | 2.1 | 5.3 |
|  | Aug | 2.4 | 2.2 | -3.1 |
|  | Sep | 2.5 | 2.5 | 8.6 |
|  | Oct | 2.9 | 2.4 | 8.2 |
|  | Nov | 2.1 | 1.8 | 18.3 |
|  | Dec | 1.6 | 1.9 | 26.6 |
| Crawfordsville, IA | Jan | 2.3 | 2.8 | 11.3 |
|  | Feb | 1.5 | 1.6 | 9.1 |
|  | Mar | 2.0 | 1.8 | 3.6 |
|  | Apr | 2.2 | 1.8 | 9.0 |
|  | May | 1.9 | 1.6 | 9.6 |
|  | Jun | 2.2 | 1.8 | 4.3 |
|  | Jul | 2.1 | 1.9 | -2.0 |
|  | Aug | 2.3 | 2.1 | -13.1 |
|  | Sep | 2.9 | 2.4 | -1.8 |
|  | Oct | 2.8 | 2.4 | 3.6 |
|  | Nov | 2.3 | 2.0 | 9.5 |
|  | Dec | 2.1 | 2.6 | 1.6 |
| Mead, NE | Jan | 1.9 | 2.3 | 13.4 |
|  | Feb | 1.4 | 1.3 | 8.8 |
|  | Mar | 1.8 | 1.7 | -0.6 |
|  | Apr | 2.0 | 1.8 | 9.7 |
|  | May | 2.0 | 1.7 | 7.4 |
|  | Jun | 2.3 | 2.0 | 2.7 |
|  | Jul | 2.1 | 2.0 | 1.1 |
|  | Aug | 2.5 | 2.2 | -8.7 |
|  | Sep | 2.9 | 2.5 | 2.3 |
|  | Oct | 2.8 | 2.5 | 0.5 |
|  | Nov | 2.1 | 1.9 | 8.0 |
|  | Dec | 2.0 | 2.4 | 17.8 |
| Ames, IA | Jan | 2.2 | 2.6 | 14.9 |
|  | Feb | 1.5 | 1.6 | 9.5 |
|  | Mar | 2.0 | 1.8 | 2.8 |
|  | Apr | 2.1 | 1.8 | 8.8 |
|  | May | 1.9 | 1.6 | 7.7 |
|  | Jun | 2.2 | 1.9 | 10.3 |
|  | Jul | 2.1 | 1.9 | -2.6 |
|  | Aug | 2.4 | 2.1 | -9.9 |
|  | Sep | 2.9 | 2.5 | -3.2 |
|  | Oct | 2.8 | 2.5 | 1.8 |
|  | Nov | 2.2 | 2.0 | 7.9 |
|  | Dec | 2.1 | 2.6 | 9.7 |
| Atkinson, NE | Jan | 1.8 | 2.3 | 17.5 |
|  | Feb | 1.3 | 1.3 | 9.2 |
|  | Mar | 1.7 | 1.8 | 8.0 |
|  | Apr | 2.1 | 1.8 | 7.5 |
|  | May | 2.0 | 1.7 | 4.3 |
|  | Jun | 2.3 | 2.0 | 7.0 |
|  | Jul | 2.2 | 2.1 | 2.1 |
|  | Aug | 2.6 | 2.3 | -11.0 |
|  | Sep | 2.9 | 2.5 | 4.1 |
|  | Oct | 3.0 | 2.4 | -5.7 |
|  | Nov | 2.3 | 1.9 | -2.3 |
|  | Dec | 1.9 | 2.4 | 29.0 |
| Nashua, IA | Jan | 2.3 | 3.0 | 17.7 |
|  | Feb | 1.6 | 2.0 | 9.9 |
|  | Mar | 2.1 | 2.0 | 6.4 |
|  | Apr | 2.3 | 1.8 | 10.9 |
|  | May | 1.9 | 1.5 | 5.3 |
|  | Jun | 2.3 | 1.8 | 5.3 |
|  | Jul | 2.3 | 1.9 | -4.6 |
|  | Aug | 2.5 | 2.1 | -6.5 |
|  | Sep | 2.9 | 2.5 | -1.2 |
|  | Oct | 2.8 | 2.5 | 5.0 |
|  | Nov | 2.4 | 2.1 | 5.6 |
|  | Dec | 2.2 | 3.0 | 9.4 |
| Brookings, SD | Jan | 2.1 | 3.0 | 15.9 |
|  | Feb | 1.6 | 2.1 | 2.1 |
|  | Mar | 2.1 | 2.1 | 12.4 |
|  | Apr | 2.5 | 1.9 | 8.4 |
|  | May | 1.9 | 1.5 | 6.2 |
|  | Jun | 2.3 | 1.9 | 8.1 |
|  | Jul | 2.4 | 2.1 | -5.2 |
|  | Aug | 2.8 | 2.4 | -8.5 |
|  | Sep | 3.0 | 2.4 | -1.8 |
|  | Oct | 2.9 | 2.4 | -5.1 |
|  | Nov | 2.4 | 2.1 | -1.6 |
|  | Dec | 2.2 | 3.2 | 20.9 |

**
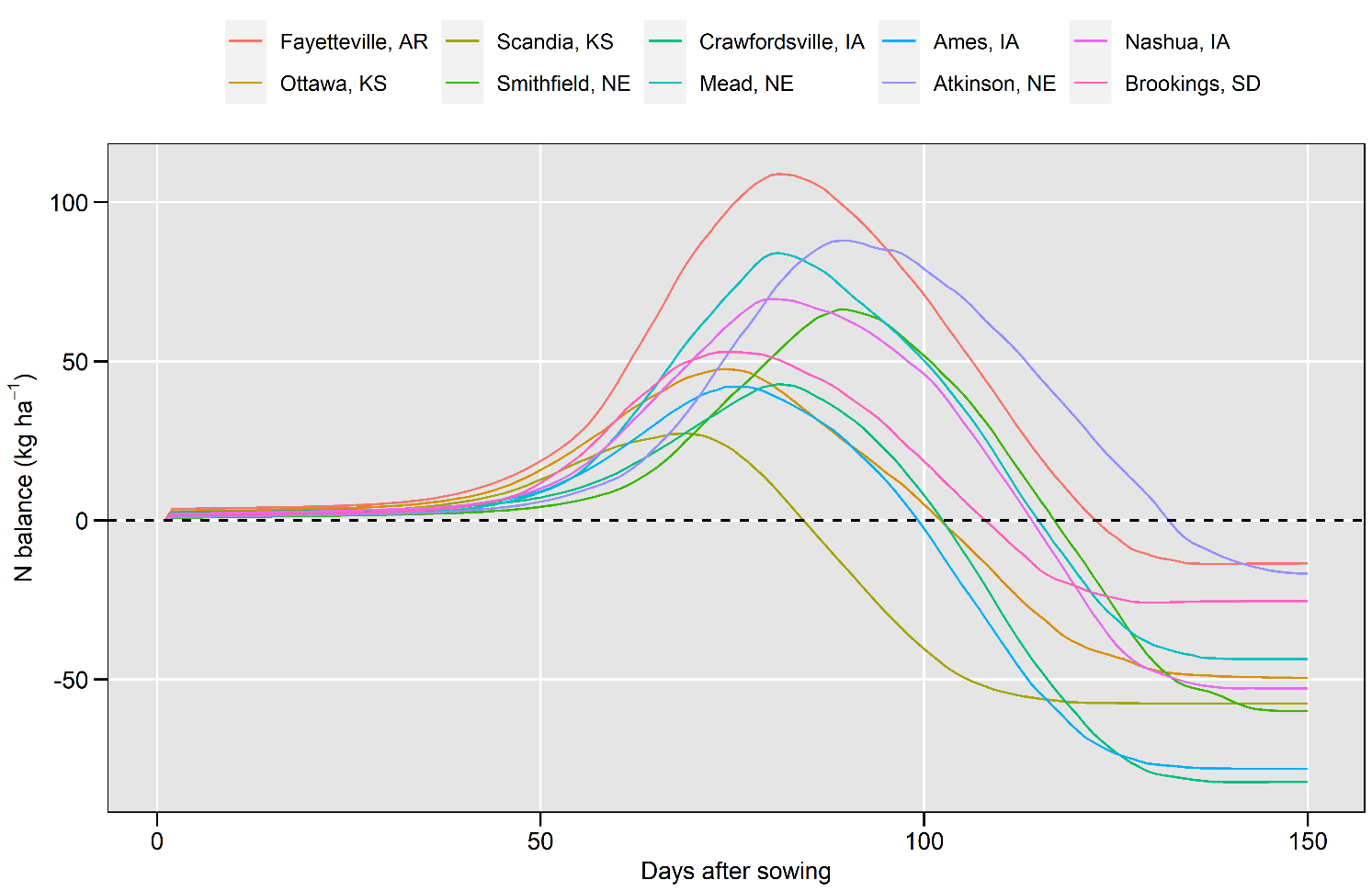
**

**Supplementary Figure 2**. N balance (BNF + N fertilization - seed N removal) during the soybean growing season for 10 locations in the US Corn Belt. The values are averages over 25-yr simulations.


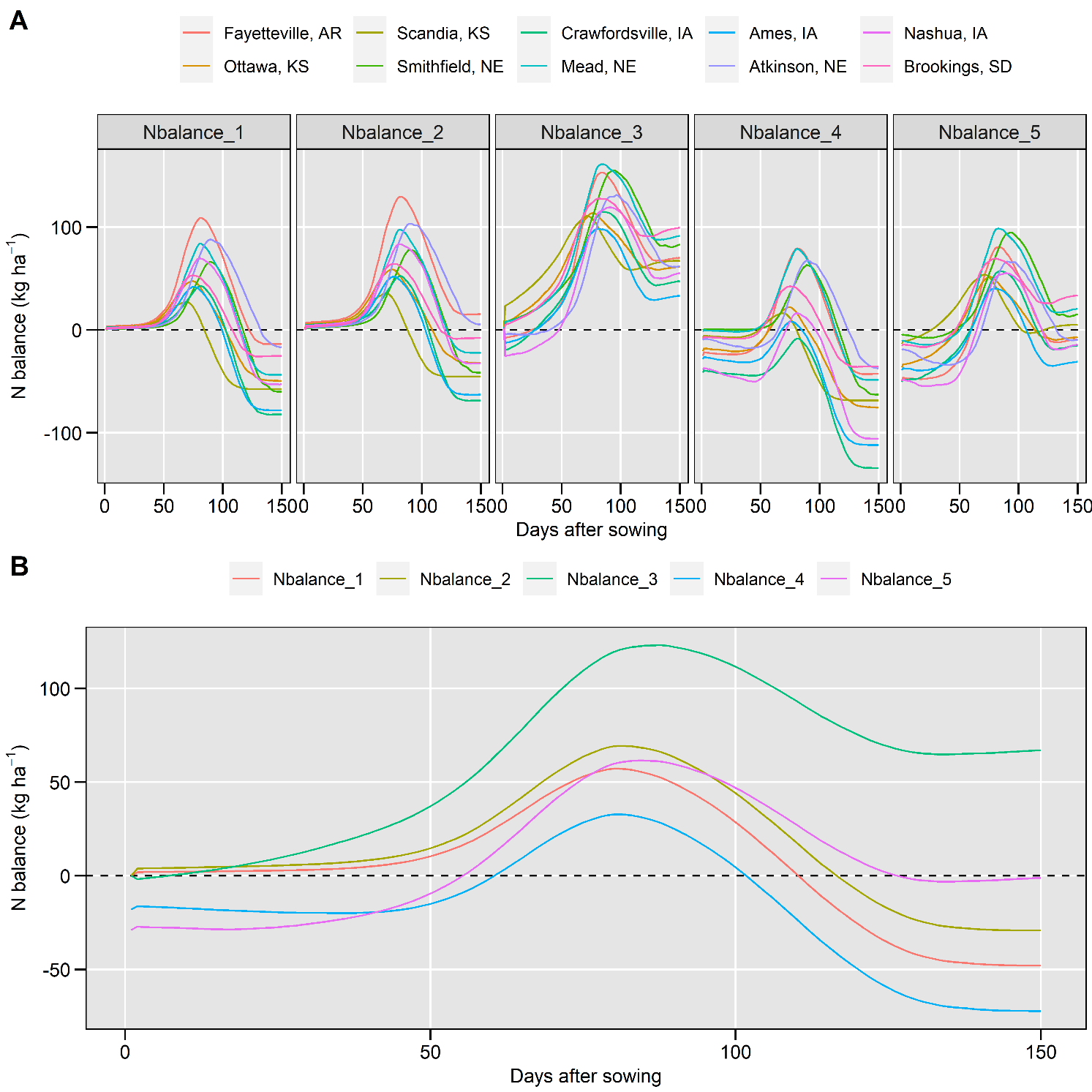


**Supplementary Figure 3**. N balance during the soybean growing season calculated using five different ways. Values are shown by location (A) and average across all locations (B).

Nbalance_1 = above ground BNF + N fertilization – seed N removal

Nbalance_2 = total BNF + N fertilization – seed N removal

Nbalance_3 = total BNF + N fertilization + N gross mineralization – seed N removal – total N loss (denitrification + leaching)

Nbalance_4 = above ground BNF + N fertilization - seed N removal – total N loss

Nbalance_5 = above ground BNF + N fertilization + N net mineralization – seed N removal – total N loss


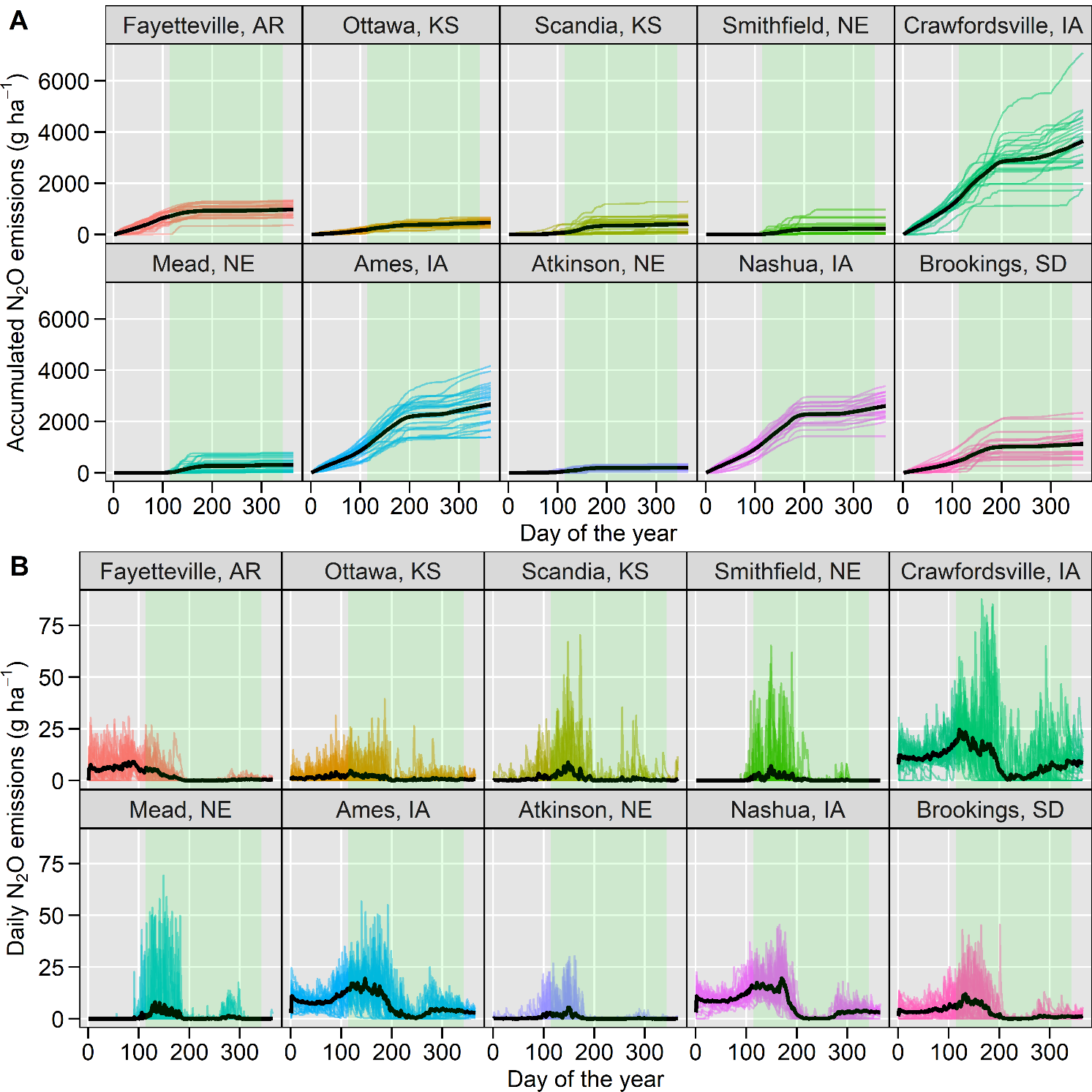


**Supplementary Figure 4**. Daily (A) and cumulative (B) N_2_O emissions over the year for 10 locations across the US Corn Belt. Black lines are averages from 25 years and colored lines show the variability across weather years. The shaded area represents the soybean growing period (covering all location-year-management combinations)

**
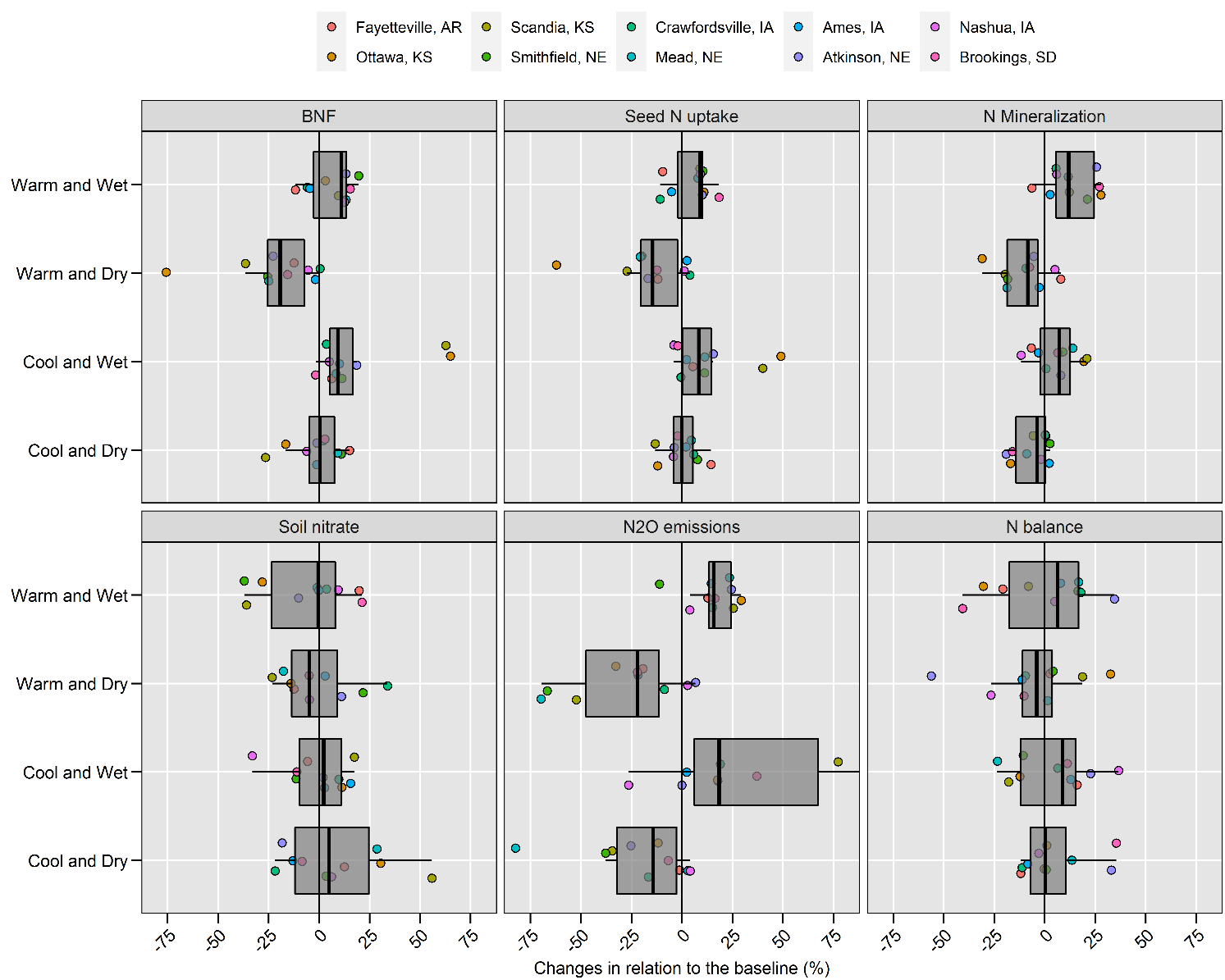
**

**Supplementary Figure 5**. Changes in soybean above ground BNF, seed N uptake, N net mineralization, Soil nitrate, N_2_O emissions and N balance at harvest time under cool-dry, cool-wet, warm-dry and warm-wet weather conditions. Grouping was performed from the 25-yr historical weather dataset (Figure 1B in the manuscript). Average values from all the 25-yrs were considered as baseline conditions.

**Supplementary Table 6.** Changes in soybean above ground N fixation (BNF), seed N accumulation (Seed N), N net mineralization (NetMin), soil nitrate (0-30 cm), grain yield at 0% moisture (Yield), N_2_O emissions and N balance under various management practices and climate change scenarios in relation to baseline conditions (%). Values are averages from 10 locations and 25 simulation years at crop harvest date.

| N° | Type of scenario | Scenario^1^ | BNF | Seed N | NetMin | Soil Nitrate | Yield | N_2_O emissions | N balance |
| --- | --- | --- | --- | --- | --- | --- | --- | --- | --- |
| 1 | Climate change | RainChange | 0.5 | -0.1 | -0.8 | -3.2 | 0.1 | 2.2 | 2.4 |
| 2 |  | TempChange | -31.7 | -19.5 | 23.7 | 34.2 | -19.1 | 18.6 | -20.4 |
| 3 |  | Rain*Temp | -30.7 | -19.1 | 22.7 | 29.8 | -18.6 | 21.9 | -18.3 |
| 4 | Nitrogen management | FerSpring | -9.0 | 1.3 | -1.3 | 10.8 | 1.0 | 40.7 | 26.5 |
| 5 |  | FerR3 | -21.3 | 3.1 | -2.1 | 40.3 | 2.0 | 14.0 | 39.8 |
| 6 |  | +LeftoverN | -23.0 | 3.1 | -2.4 | 42.2 | 2.1 | 93.1 | -91.1 |
| 7 |  | - LeftoverN | 13.7 | -3.3 | 3.3 | -12.9 | -2.9 | -49.9 | 60.7 |
| 8 | Residue management and CN ratio | +ResCN | 8.2 | 2.3 | -11.7 | -20.9 | 2.2 | 14.6 | 17.4 |
| 9 |  | -ResCN | -10.5 | 0.1 | 33.6 | 14.5 | -0.1 | 12.6 | -35.7 |
| 10 |  | +Residue | 25.2 | 2.7 | -52.1 | -29.6 | 2.6 | -2.2 | 72.6 |
| 11 |  | -Residue | -19.0 | -2.1 | 45.6 | 14.3 | -2.3 | 13.4 | -54.5 |
| 12 |  | FullTillage | -9.8 | -3.5 | 5.5 | -2.6 | -3.4 | -12.2 | -17.2 |
| 13 | Plant management | +Density | 7.0 | 1.1 | -5.5 | -8.2 | 1.2 | -4.6 | 18.4 |
| 14 |  | -Density | -10.8 | -5.8 | 2.2 | 8.0 | -6.1 | 4.8 | -10.7 |
| 15 |  | EarlySow | 9.3 | 4.2 | -6.7 | -4.3 | 4.7 | -3.9 | 12.3 |
| 16 |  | LateSow | -12.0 | -6.6 | 6.9 | 4.0 | -7.0 | 6.8 | -10.3 |
| 17 | Seed protein | +SeedProtein | 5.7 | 5.3 | 0.3 | -3.7 | -0.5 | -0.7 | -4.7 |
| 18 |  | -SeedProtein | -5.8 | -5.6 | -0.4 | 4.2 | 0.5 | 0.9 | 5.6 |
| 19 | Soil organic carbon | +SOC | -4.9 | 0.7 | 24.5 | 19.2 | 0.5 | 17.9 | -19.7 |
| 20 |  | -SOC | 5.0 | -0.8 | -24.7 | -18.3 | -0.6 | -15.9 | 20.5 |

^1^ Full description of the acronyms can be found in Table 1.


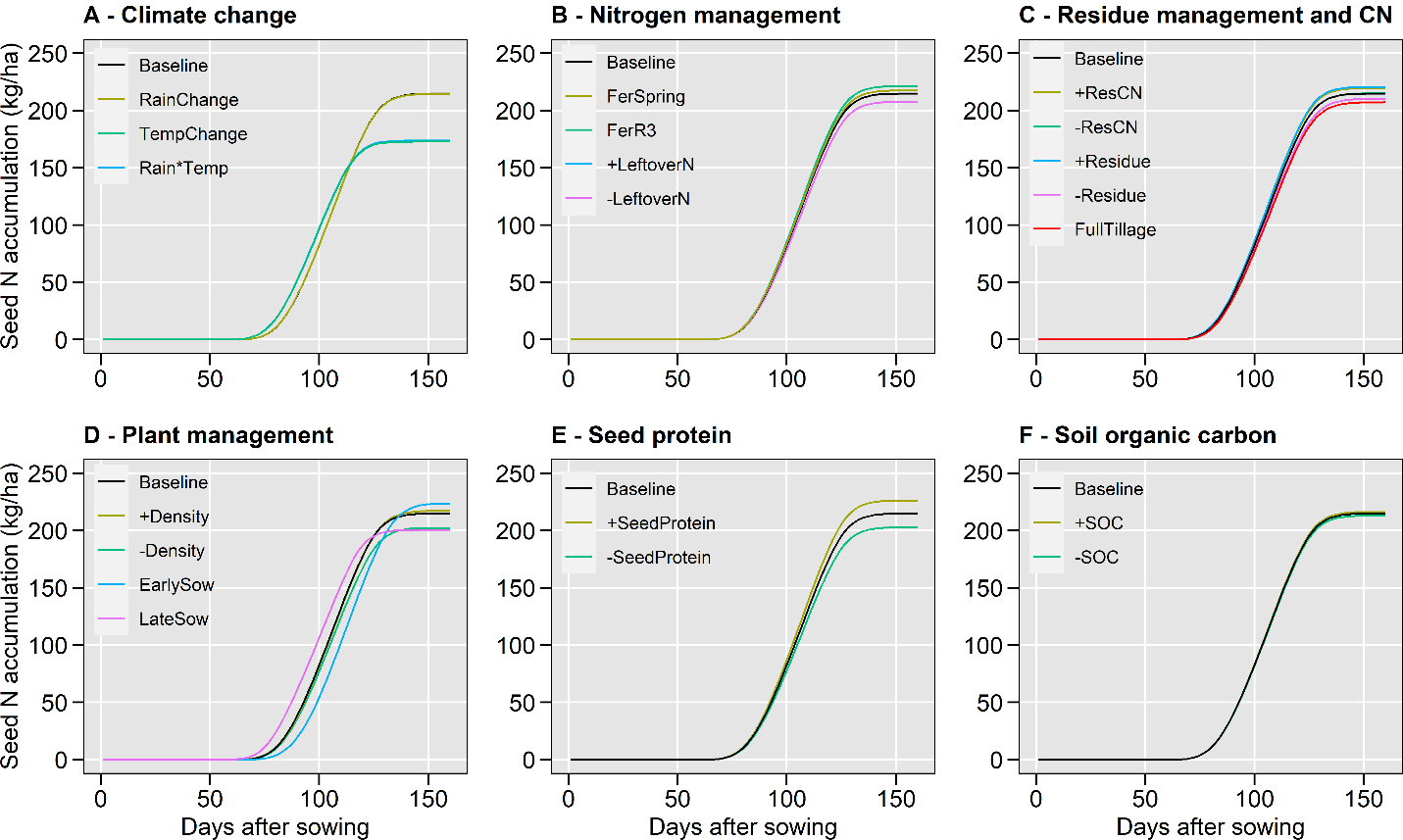


**Supplementary Figure 6**. GxExM effects on seed N accumulation during the soybean growing season for different scenarios including climate change (A), nitrogen management (B), residue management and quality (C), plant management (D), seed protein (E) and soil organic carbon (F). Values were averaged over 10 locations and 25-years per locations.


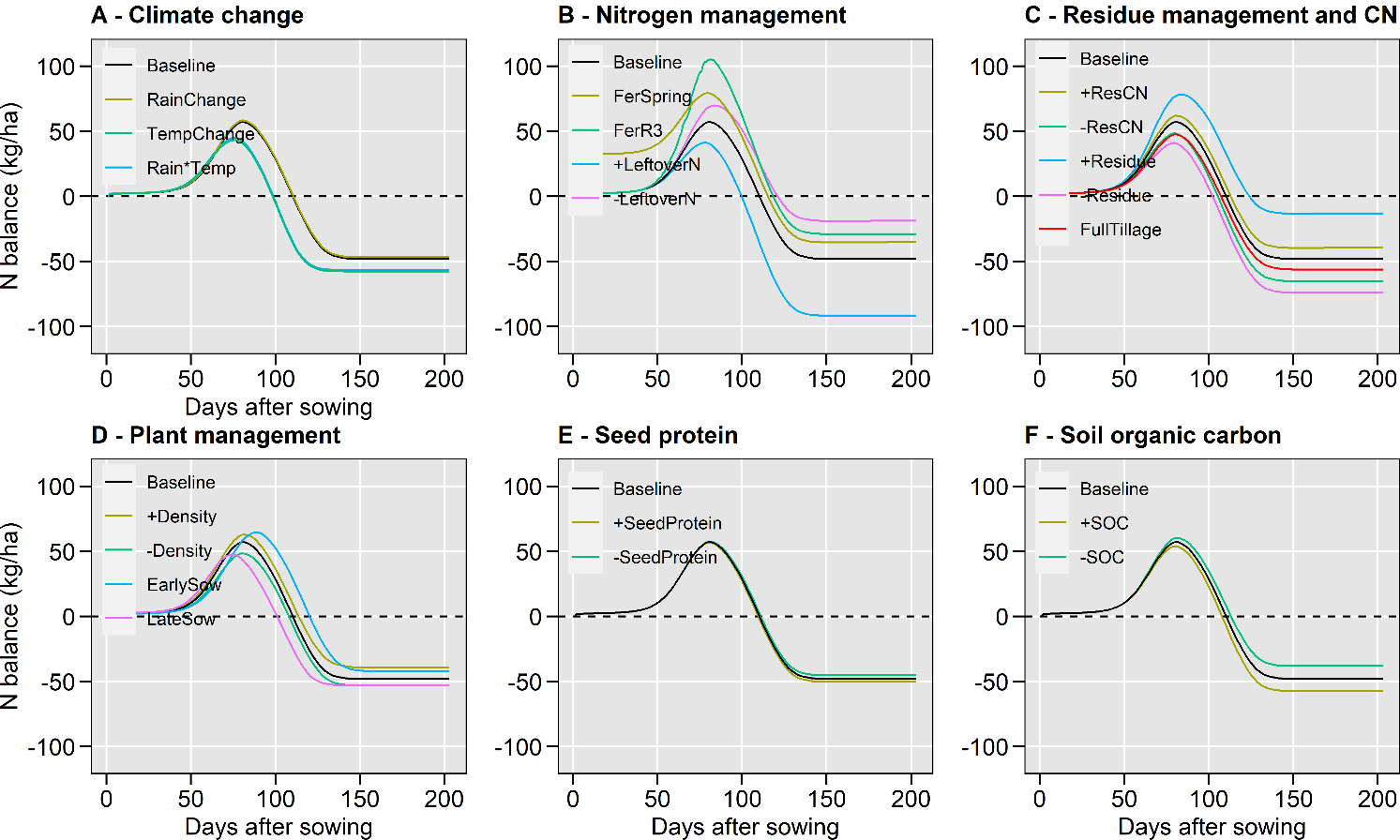


**Supplementary Figure 7**. GxExM effects on soybean N balance (BNF + N fertilization - seed N removal) during the soybean growing season for different scenarios including climate change (A), nitrogen management (B), residue management and quality (C), plant management (D), seed protein (E) and soil organic carbon (F). Values were averaged over 10 locations and 25-years per locations.

**
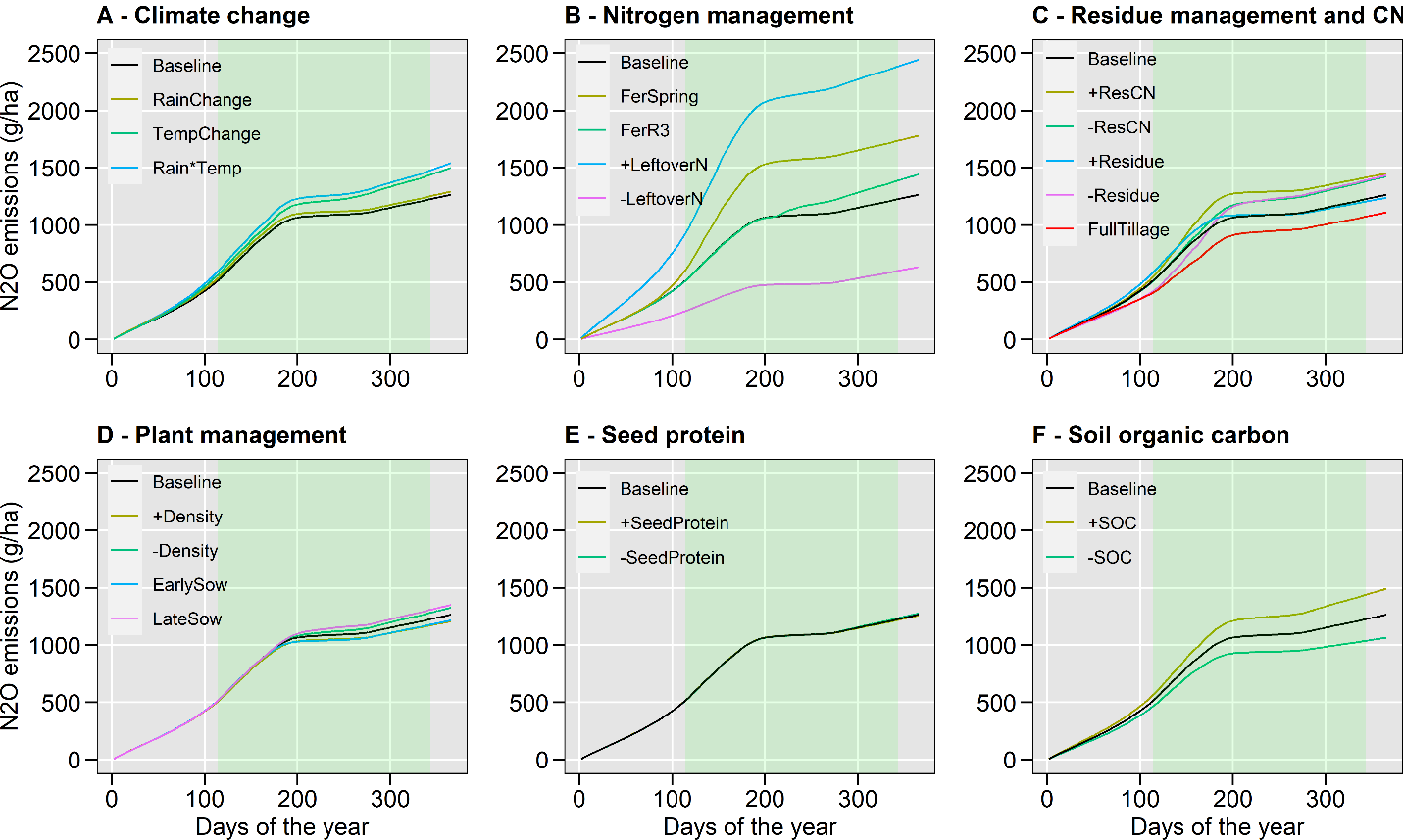
**

**Supplementary Figure 8**. GxExM effects on N_2_O emissions during the soybean growing season for different scenarios including climate change (A), nitrogen management (B), residue management and quality (C), plant management (D), seed protein (E) and soil organic carbon (F). Values were averaged over 10 locations and 25-years per locations.

**
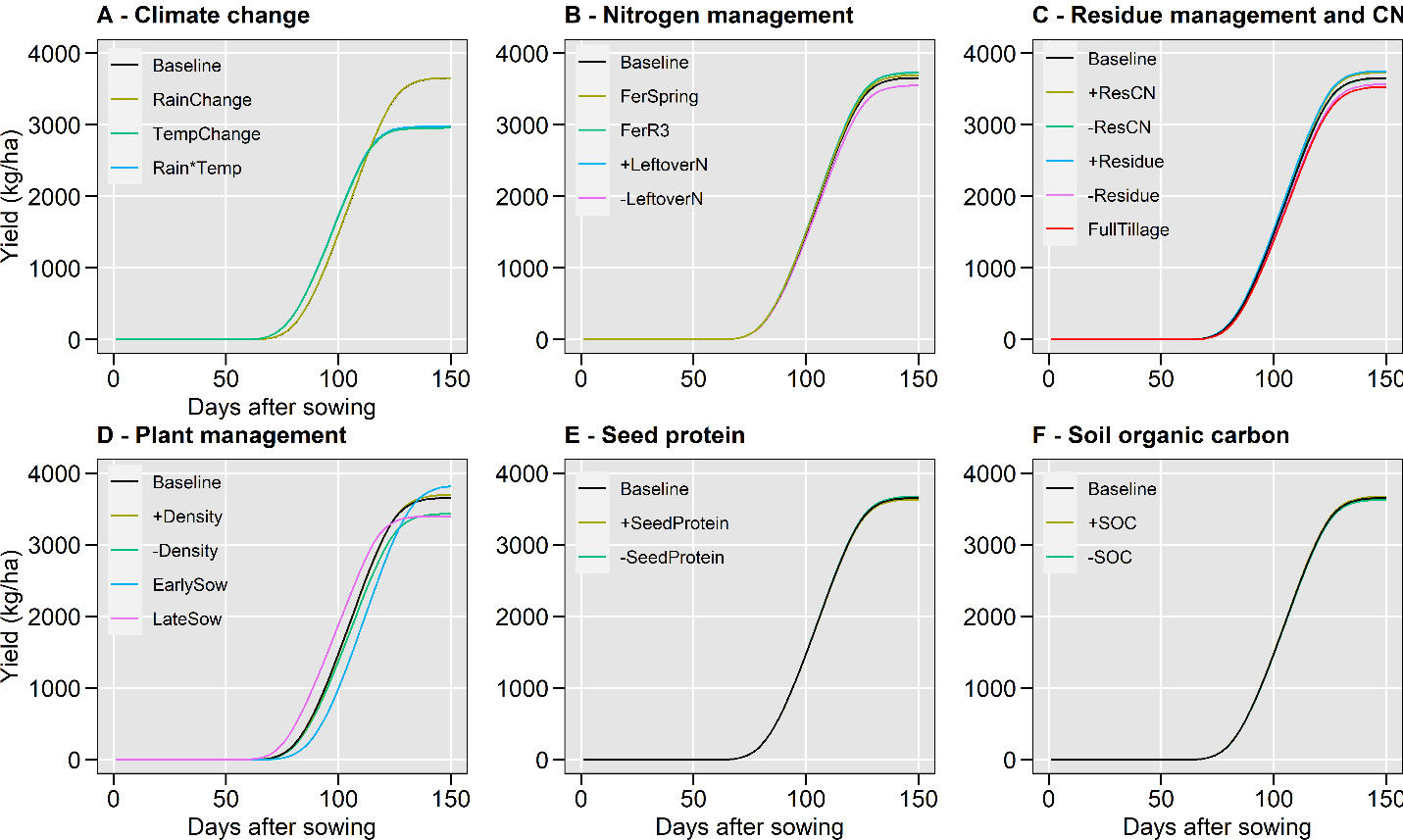
**

**Supplementary Figure 9**. GxExM effects on soybean seed yield for different scenarios including climate change (A), nitrogen management (B), residue management and quality (C), plant management (D), seed protein (E) and soil organic carbon (F). Values were averaged over 10 locations and 25-years per locations.

**
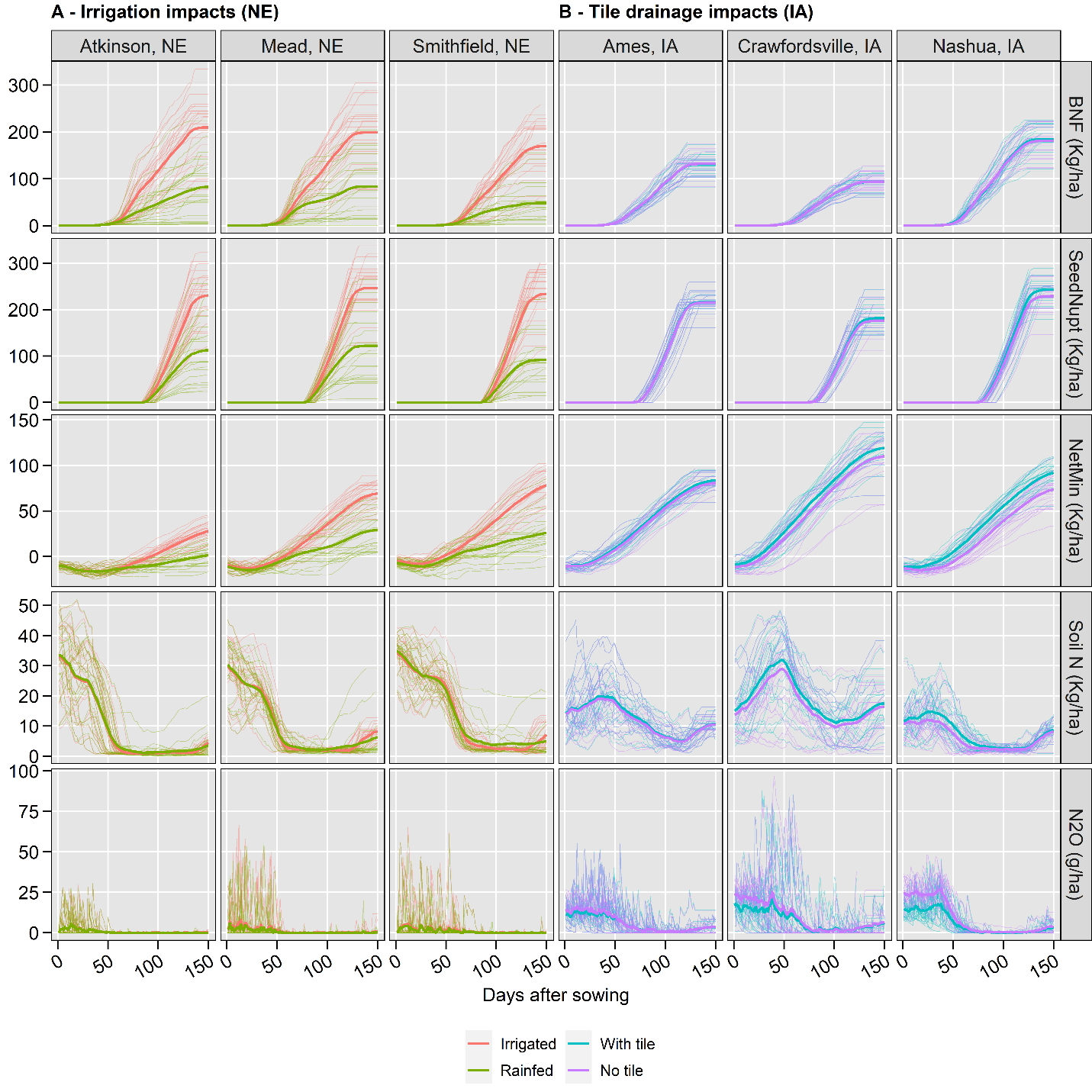
Supplementary Figure 10**. Effects of water management practices on N fixation, grain N uptake, net N mineralization and on topsoil (0-30 cm) nitrate during the soybean growing cycle. Thick lines represent the 25-year historical average while thin lines denote the individual historical years.


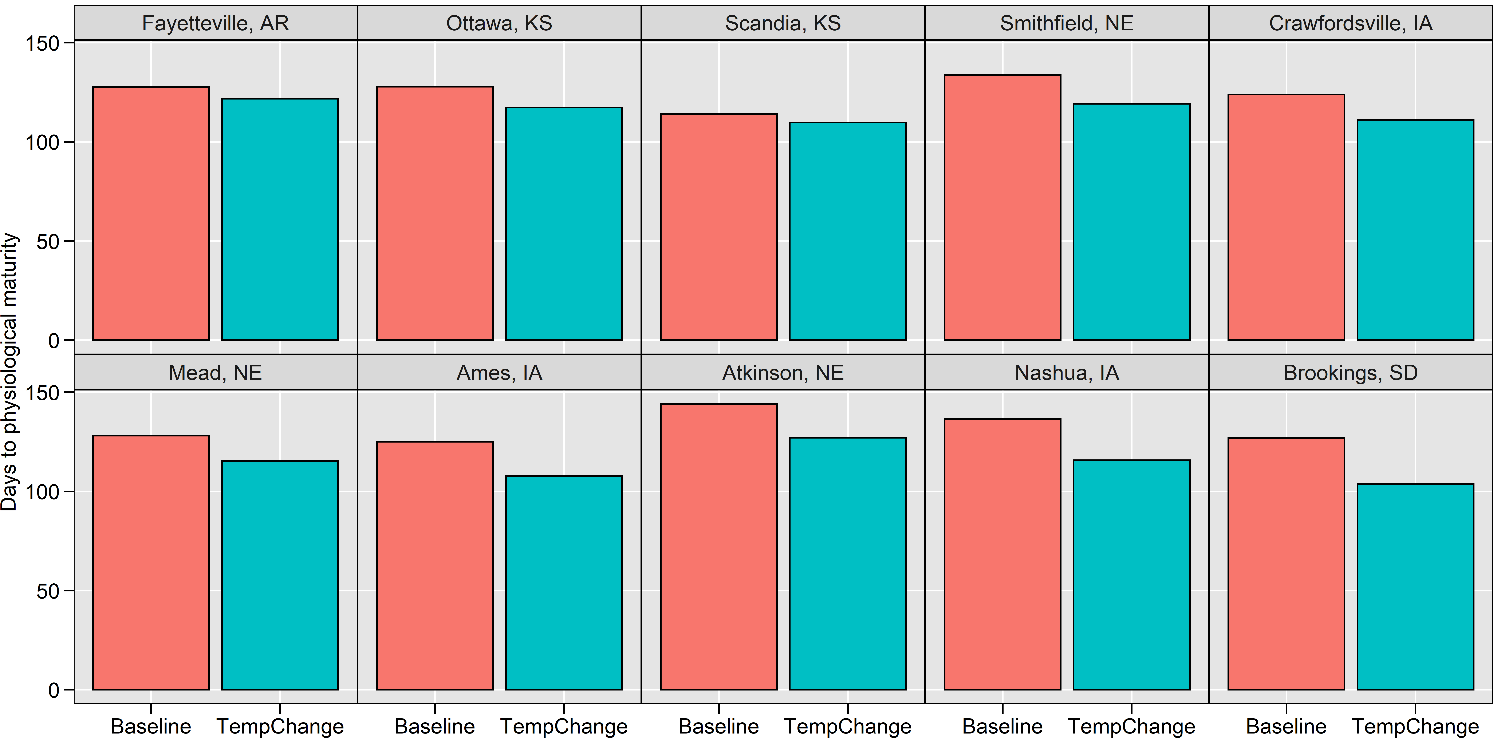


**Supplementary Figure 11.** Average number of days for soybean crop to reach physiological maturity under baseline and warming temperature (TempChange scenario


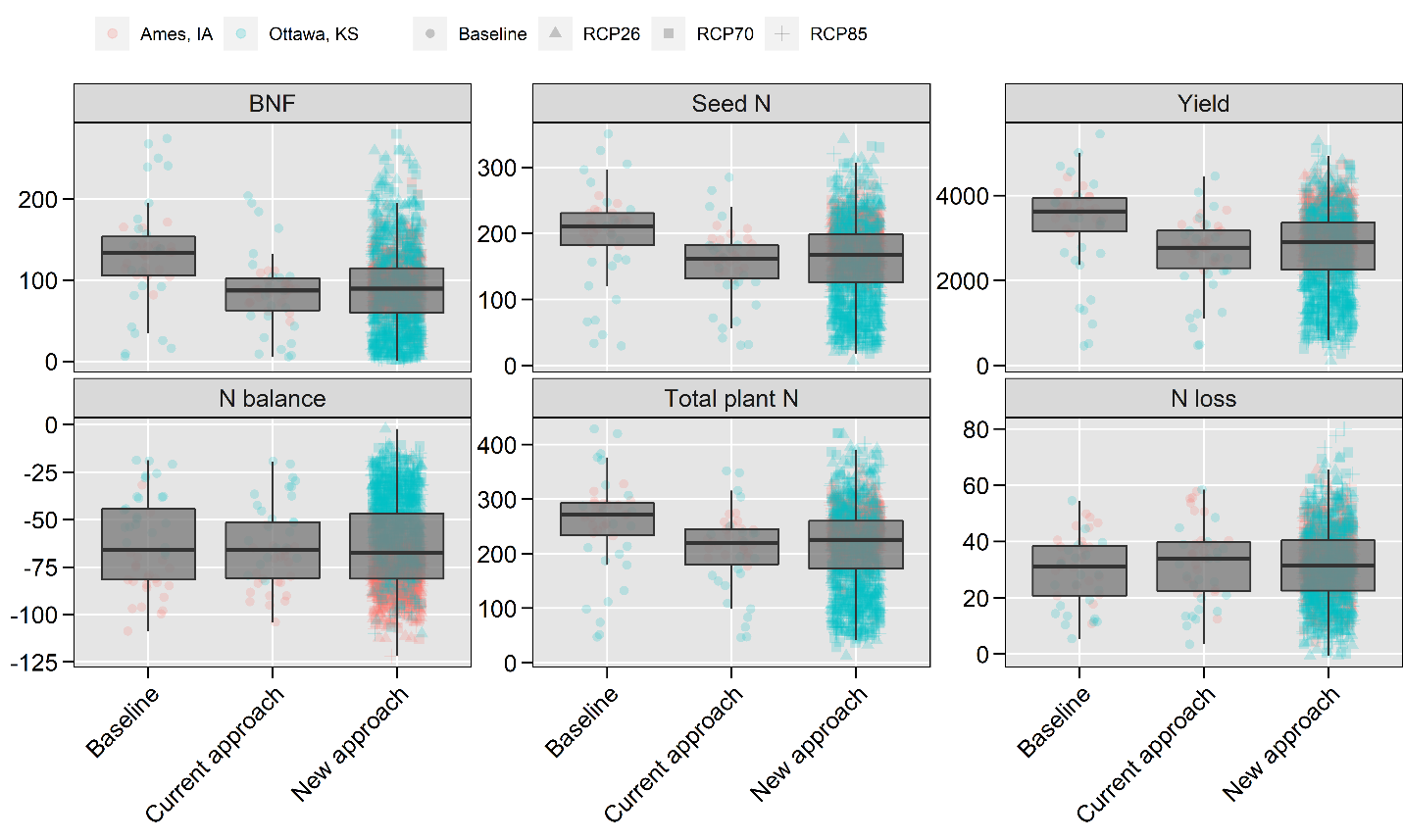


**Supplementary Figure 12.** Comparison of different N fluxes and crop yields (kg ha^-1^) by using the approach from this study (Current approach) for climate change assessments and using daily outputs from 15 GCMs-RCPs climate scenarios from 2020-2080 (New approach) in two contrasting locations.


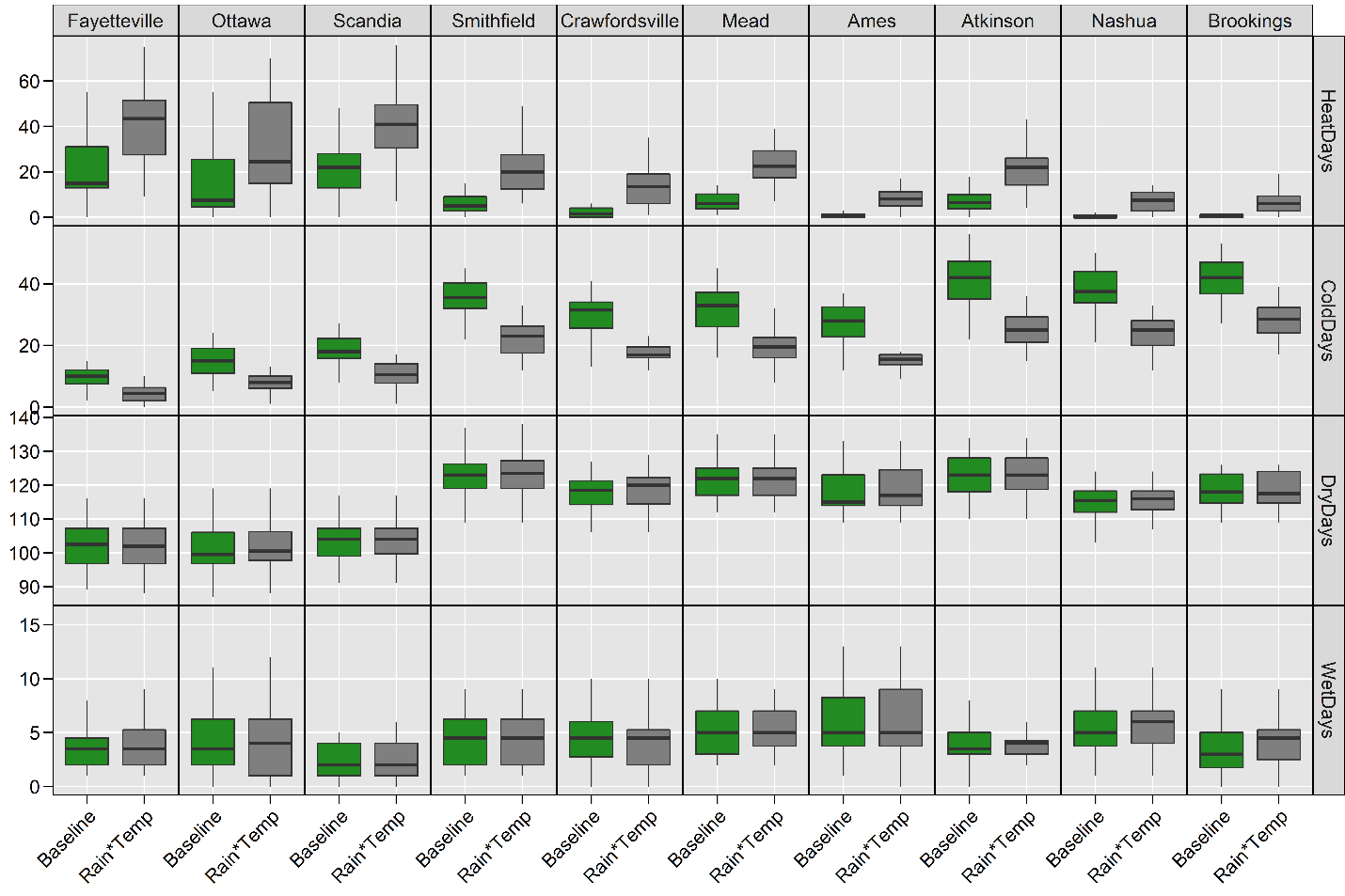


**Supplementary Figure 13.** Climate extremes (May to September) as affected by climate change scenarios. HeatDays = number of days with maximum temperature > 35°C, ColdDays = number of days with minimum temperature < 10°C, DryDays = number of days with daily rain < 2mm, WetDays = number of days with daily rain < 25mm.
